# *Cdk8* is required for establishment of H3K27me3 and gene repression by *Xist* and mouse development

**DOI:** 10.1242/dev.175141

**Published:** 2020-06-11

**Authors:** Andreas Postlmayr, Charles Etienne Dumeau, Anton Wutz

**Affiliations:** D-BIOL, Institute of Molecular Health Sciences, Swiss Federal Institute of Technology, ETH Hönggerberg, HPL E12, Otto-Stern-Weg 7, 8049 Zurich, Switzerland

**Keywords:** Cdk8, Cyclin dependent kinase, Xist, X inactivation, Polycomb, Gene regulation

## Abstract

We previously identified the cyclin dependent kinase *Cdk8* as a putative silencing factor for *Xist*. To investigate its role in X inactivation, we engineered a *Cdk8* mutation in mouse embryonic stem cells (ESCs) carrying an inducible system for studying *Xist* function. We found that *Xist* repressed X-linked genes at half of the expression level in *Cdk8* mutant cells, whereas they were almost completely silenced in the controls. Lack of *Cdk8* impaired Ezh2 recruitment and the establishment of histone H3 lysine 27 tri-methylation but not PRC1 recruitment by *Xist*. Transgenic expression of wild-type but not catalytically inactive *Cdk8* restored efficient gene repression and PRC2 recruitment. Mutation of the paralogous kinase *Cdk19* did not affect *Xist* function, and combined mutations of *Cdk8* and *Cdk19* resembled the *Cdk8* mutation. In mice, a *Cdk8* mutation caused post-implantation lethality. We observed that homozygous *Cdk8* mutant female embryos showed a greater developmental delay than males on day 10.5. Together with the inefficient repression of X-linked genes in differentiating *Cdk8* mutant female ESCs, these data show a requirement for *Cdk8* in the initiation of X inactivation.

## Abstract (precis)

**Summary:** Mutagenesis studies enabled the functional characterization of the cyclin-dependent kinase Cdk8 in X chromosome inactivation, and showed that Cdk8 is required for efficient gene repression and PRC2 recruitment by Xist.

## INTRODUCTION

Mammals achieve dosage compensation for the different number of X chromosomes in male and female cells by silencing of the transcription of one of the two X chromosomes (Lyon, 1962). X chromosome inactivation (XCI) is initiated by the expression of the long noncoding *Xist* RNA, which localizes to the future inactive X chromosome (Xi), and triggers chromatin modifications and gene repression in an almost chromosome-wide manner (Galupa and Heard, 2018). The process of chromosomal silencing has been studied in mice and mouse embryonic stem cells (ESCs). Initially, in female mouse embryos, *Xist* is expressed from the paternally inherited X chromosome at the four-cell stage and imprinted XCI is observed. In the cells of the inner cell mass of the blastocyst that form the epiblast lineage, imprinted XCI is reversed and two active X chromosomes are present in the female embryos. Subsequently, the inactivation of either the maternal or paternal inherited X chromosome is initiated in the embryo at day 5.5. The initiation of random XCI has also been extensively studied in female mouse ESCs, which initially possess two active X chromosomes and undergo XCI upon differentiation. *Xist* is expressed from and localizes to the future Xi and establishes a domain of repressive chromatin (Chaumeil et al., 2008). Initially, this repressive compartment is spatially separated from genes, which remain expressed and are silenced by a separate mechanism that requires A repeat sequences of *Xist* RNA, as well as the RNA-binding protein Spen (Chu et al., 2015; McHugh et al., 2015; Monfort et al., 2015; Wutz et al., 2002). The recruitment of polycomb proteins by *Xist* induces chromosome-wide histone modifications and this recruitment requires hnRNPK (Chu et al., 2015; Pintacuda et al., 2017), and the polycomb group proteins Pcgf3 and Pcgf5 (Almeida et al., 2017). It is thought that hnRNPK binds to *Xist* repeat B, and through the recruitment of Ring1b- and Ring1A-containing complexes, leads to ubiquitylation of histone H2A lysine 119 (H2AK119ub). This initial chromatin modification triggers the recruitment of additional polycomb repressive complex 1 (PRC1) and PRC2 (Almeida et al., 2017). PRC2 recruitment in turn catalyses trimethylation of histone H3 lysine 27 (H3K27me3). Multiple interactions of different polycomb group proteins with H2AK119ub and H3K27me3 are thought to cause an enrichment of a large number of polycomb proteins on the Xi, and to contribute to the establishment of a domain of repressive chromatin. Recent biochemical and genetic studies have identified a number of additional factors that contribute to the process of X inactivation (Chu et al., 2015; McHugh et al., 2015; Moindrot et al., 2015; Ridings-Figueroa et al., 2017). The function of *Xist* in chromosomal silencing has also been studied by forced expression of *Xist* in male ESCs, allowing the uncoupling of the formation of a silent chromosome from the complex regulation of the *Xist* gene (Chaumeil et al., 2008; Chu et al., 2015; McHugh et al., 2015; Plath et al., 2003; Wutz and Jaenisch, 2000). We have previously used an inducible *Xist* expression system for a genetic screen in mouse haploid ESCs for identifying mutations that prevent the repression of X-linked genes by forced *Xist* expression (Monfort et al., 2015). Among the candidate genes identified as required for *Xist* function is the atypical cyclin-dependent kinase *Cdk8*.

Cdk8 interacts with the mediator complex, which has a central role in the regulation of transcription. Biochemically, Cdk8 associates with a kinase module of the mediator that contains the Cdk8, CycC, Med12 and Med13 proteins (Clark et al., 2015; Jeronimo and Robert, 2017). It is thought that *Cdk8* has a role in fine-tuning transcription and can exert repressive, as well as activating, functions on mediator-regulated gene expression (Gobert et al., 2010; Papadopoulou et al., 2016). Negative regulation of TFIIH activity through the phosphorylation of cyclin H by Cdk8, has been demonstrated (Akoulitchev et al., 2000). However, it is likely that *Cdk8* acts through additional mechanisms that remain to be fully understood (Andrau et al., 2006; Fukasawa et al., 2015). In mouse pluripotent cells, the Med12/Med13 module associates with PRC1-binding sites and H2AK119ub (Papadopoulou et al., 2016). However, Cdk8 appears to be stably bound to only a small subset of these sites. In addition, Cdk8 binding is associated with active genes. Recent evidence from the inhibition of Cdk8 kinase activity in lymphoma cells suggests a repressive function for *Cdk8* on genes that are regulated by super-enhancers (Pelish et al., 2015). The function of mediator kinases is difficult to assess, as a homologous complex containing Cdk19, CycC, Med12L and Med13L is also present in vertebrates (Bourbon 2008; Daniels et al., 2013). It is thought that Cdk8 and Cdk19 form mutually exclusive complexes that might have overlapping functions. Cdk8 and Cdk19 share 97% sequence homology in their catalytic domain and differ at their C-termini (Sato et al., 2004). Here, we investigate the function of the mediator kinases for gene repression by *Xist*.

## RESULTS

### *Cdk8* is required for efficient gene repression by *Xist*

**Fig. 1.**
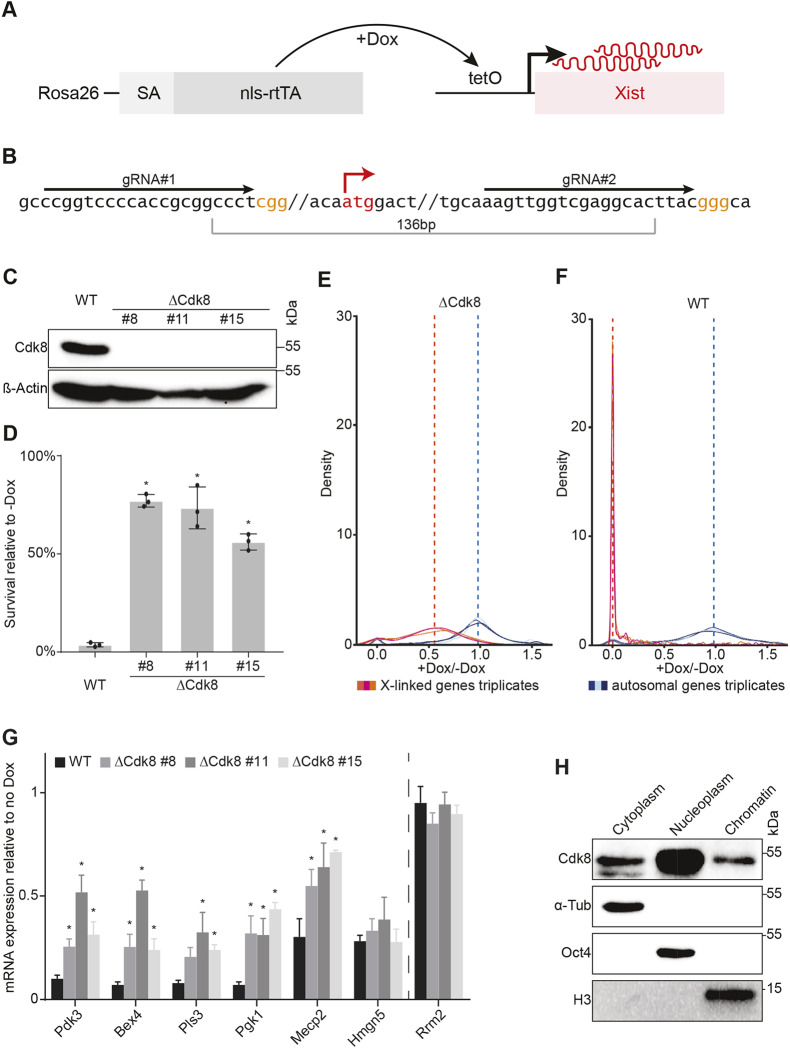
**Loss of *Cdk8* impairs X-linked gene silencing by *Xist*.** (A) Schematic of the *Xist* expression system in HATX3 ESCs. The nls-rtTA transactivator binds an inducible tetO promoter at the start site of the *Xist* gene. Doxycycline addition leads to *Xist* expression. nls-rtTA, nuclear localization signal-reverse tetracycline-controlled transactivator; tetO, tetracycline operator; Dox, doxycycline; SA, splice acceptor; Rosa26, genomic locus of nls-rtTA integration. (B) CRISPR/Cas9 strategy to engineer ΔCdk8 HATX3 ESCs; two gRNAs were designed to excise 136 bp, including the Cdk8 start codon. Yellow, PAM sequence; red, start codon; black arrows, gRNA target sequences. (C) Immunoblot confirming the absence of Cdk8 protein in ΔCdk8 ESC clones 8, 11 and 15. WT represents parental HATX3 ESCs. β-Actin, loading control. (D) Single cell survival assay for measuring *Xist* function. The ratio of survival of WT and ΔCdk8 after *Xist* induction relative to uninduced conditions is shown. The experiments were performed in triplicate. Data are mean±s.d.; asterisk indicates significant changes relative to WT (*P*<0.05). (E,F) RNAseq data representation of expression ratio after 48 h of *Xist* expression relative to untreated conditions for ΔCdk8 cells (E) and wild-type cells (F). Red curves, ratio of X-linked gene expression; blue curves, ratio of autosomal gene expression; dashed lines, median values. (G) qRT-PCR validation of differentially regulated X-linked genes in ΔCdk8 compared with WT ESCs after 48 h of *Xist* expression. *Rrm2* serves as autosomal control. Expression levels were normalised to *Gapdh* and are relative to uninduced conditions. The experiments were performed in triplicate. Data are mean±s.d.; asterisk indicates significant changes relative to WT (*P*<0.05). (H) Western blot analysis of cell fractionation showing localisation of Cdk8, α-Tubulin (cytoplasmic marker), Oct4 (nucleoplasmic marker) and histone H3 (chromatin fraction).

For investigating the effect of *Cdk8* on *Xist* function, we engineered a mutation of *Cdk8* in mouse HATX3 ESCs that carry a doxycycline-inducible *Xist* allele (Fig. 1A). HATX3 ESCs were established from haploid mouse embryos (Monfort et al., 2015) and became diploid in culture. Induction of *Xist* causes repression of X-linked genes and leads to cell death. A small deletion that includes the start codon was introduced into the *Cdk8* locus (Fig. 1B). Several clones were identified to carry homozygous mutations and the absence of Cdk8 protein was confirmed by western blot analysis (Fig. 1C). Three independent clones were selected for further analysis. Sequencing of the genomic locus revealed that a small insertion had occurred in one clone (Fig. S1A) that might explain residual transcript in this clone (Fig. S1B). However, Cdk8 protein was undetectable by western blot analysis. Induction of *Xist* expression in parental HATX3 and *Cdk8* mutant ESCs (ΔCdk8) resulted in cell loss. To quantify the magnitude of cell loss, we applied a single cell assay, whereby individual cells were deposited into 96-well plates and the number of colonies were counted after 14 days in the presence or absence of doxycycline. The ratio of the number of colonies obtained in the presence of doxycycline relative to the absence of doxycycline was calculated to determine the percentage of survival after *Xist* induction (Fig. S1C). This assay revealed a substantial increase in the survival of cells lacking *Cdk8* (Fig. 1D). This suggested a potential requirement for *Cdk8* in *Xist* function. To further assess whether this survival was caused by changes in X-linked gene repression, we performed RNAseq analysis of *Cdk8* mutant and wild-type cells after 48 h of *Xist* induction, and in uninduced conditions. We quantified expression changes as the ratio of gene expression in induced conditions relative to uninduced conditions (Fig. 1E,F). Residual expression of X-linked genes in wild-type cells expressing *Xist*, was strongly reduced compared with uninduced cells (Fig. 1F). However, in cells lacking *Cdk8*, X-linked genes were on average expressed at half the level measured in uninduced conditions (Fig. 1E, Table S1). Overall, transcription was not affected by *Xist*, as shown by the unchanged expression of autosomal genes (Fig. 1E,F). Furthermore, the *Cdk8* mutation did not lead to an overall change in X-linked gene expression before the induction of *Xist* (Fig. S1D). This observation suggested that *Xist* was able to repress genes in the absence of *Cdk8* but the repression was incomplete. Investigation of the gene level further showed variability over different X-linked genes (Fig. S1E), which likely reflects the different half-lives of the transcripts, as well as the efficiency of silencing. The latter has been observed before and is probably caused by multiple and gene-specific repression mechanisms acting in X inactivation (Żylicz et al., 2019). To obtain an independent confirmation, we determined the expression levels of several genes by qRT-PCR. The X-linked *Pdk3*, *Bex4*, *Pls3*, *Pgk1* and *Mecp2* genes showed higher residual expression in *Cdk8* mutant cells compared with wild-type control cells, when *Xist* was induced (Fig. 1G). In contrast, *Hmgn5* was repressed to comparable levels in *Cdk8* mutant and control cells, showing that the requirement of *Cdk8* for efficient repression was gene specific (Fig. 1G). Induction of *Xist* had no measurable effect on the autosomal *Rrm2* gene (Fig. 1G). In addition, we assessed the relative expression differences of the above genes in *Cdk8* mutant and control cells in the absence of *Xist* induction. Expression levels were comparable, which confirmed that the baseline expression in control and *Cdk8* mutant cells is comparable before the induction of *Xist* (Fig. S1F). Biochemical fractionation showed that Cdk8 is localized to the nucleus in ESCs and can be detected in the chromatin-associated fraction (Fig. 1H), which is consistent with its function in gene regulation.

### The catalytic activity of Cdk8 is required for *Xist* function

**Fig. 2.**
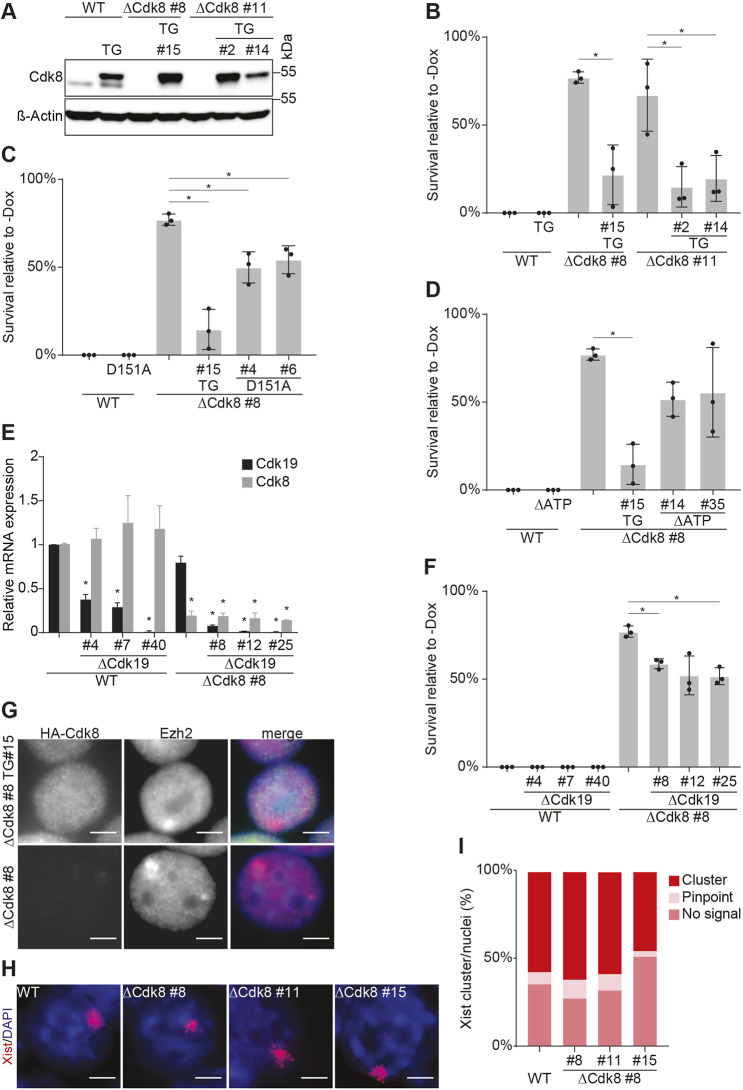
***Cdk8* kinase activity is required for *Xist* function but not *Xist* localization.** (A) Immunoblot confirming expression of *Cdk8* wild-type transgene in ΔCdk8 (clones 8 and 11) and wild-type ESCs. Transgenic HA-Cdk8 shows a higher molecular weight. β-Actin, loading control. (B-D) Survival ratio after *Xist* induction (as in Fig. 1D) of ESCs expressing a wild-type *Cdk8* transgene (B), a D151A mutant *Cdk8* transgene (C) and ΔATP mutant *Cdk8* transgene (D), showing complementation of ΔCdk8 ESCs with the wild-type *Cdk8* transgene. The experiments were performed in triplicate. Data are mean±s.d.; asterisk indicates statistically significant changes (*P*<0.05). ΔCdk8#8 and ΔCdk8#8 TG are the same in all panels. (E) qRT-PCR analysis of Cdk8 and Cdk19 expression in ΔCdk19 mutant clones, ΔCdk8#8 ΔCdk19 double mutant clones and control HATX3 (WT) ESCs, using primers in *Cdk19* exons 1 and 2, and *Cdk8* exons 2 and 3. Expression is normalized using *Gapdh* and shown relative to WT. Experiments were performed in triplicate. Data are mean±s.d.; asterisk indicates statistically significant changes relative to WT (*P*<0.05). (F) Survival ratio after *Xist* induction of ΔCdk19 and ΔCdk8#8 ΔCdk19 ESCs (as in B). Experiments were performed in triplicate; data are mean±s.d.; asterisk indicates statistically significant changes (*P*<0.05). (G) Immunofluorescence analysis showing localisation of HA-Cdk8 and Ezh2 (a Xi marker). (H) *Xist* RNA FISH (red) of wild-type and ΔCdk8 ESCs. DAPI was used to stain DNA (blue). (I) Quantification of *Xist* RNA FISH showing percentage of clusters (red), pinpoint signals (light pink) and no signal (dark pink) for genotypes as indicated. The experiments were performed in triplicate; >100 nuclei counted. Scale bars: 5 μm.

To demonstrate the specificity of the *Cdk8* mutation and exclude potential off-target effects, we complemented two *Cdk8* mutant cell lines with cDNA expression constructs. Several ESC clones were isolated and Cdk8 protein expression was investigated by western blot analysis (Fig. 2A). In these complemented cells, the restoration of the function of *Xist* was evident through reduced cell survival upon *Xist* induction (Fig. 2B). Taken together, these data indicate that *Cdk8* contributes to *Xist*-induced gene repression in mouse ESCs.

To further delineate the mechanism of *Cdk8* in *Xist* function, we next investigated the requirement for Cdk8 kinase activity. We introduced two mutations into the *Cdk8* cDNA that either abolish ATP binding or the function of the proton acceptor. Both mutations are predicted to lead to a loss of catalytic activity (Akoulitchev et al., 2000; Furumoto et al., 2007; Schneider et al., 2011). We introduced these mutated versions into *Cdk8* mutant and wild-type control ESCs, and confirmed protein expression by western blot analysis (Fig. S2A,B). To assess the effect on *Xist* function, we performed cell survival assays. Whereas expression of wild-type *Cdk8* restored *Xist* function in *Cdk8* mutant cells, neither of the mutated versions of *Cdk8* were able to complement *Xist* function in *Cdk8* mutant cells (Fig. 2C,D). Furthermore, the expression of either *Cdk8* mutant cDNAs in control wild-type cells did not lead to a measurable effect on *Xist* function. Taken together, these data show that the catalytic activity of Cdk8 is required for *Xist* function in our cell system.

### *Cdk8* but not *Cdk19* contributes to *Xist* function in mouse ESCs

*Cdk19* is a paralogue of *Cdk8* that is expressed in mouse ESCs and could potentially compensate for its function in *Cdk8* mutant cells. It is thought that Cdk19 complexes with Med12L and Med13L to form a submodule of the mediator complex that is similar to that formed by Cdk8. Either Cdk8 or Cdk19 submodules can associate with different core mediator complexes. To assess whether a specific requirement for *Cdk8* exists or whether *Cdk19* could also contribute to *Xist* function, we performed an analysis of the *Cdk19* mutation in our ESC system. For this purpose, we engineered mutations in the *Cdk19* gene in wild-type HATX3 cells and *Cdk8* mutant ΔCdk8 cells. RT-PCR analysis showed a strong reduction of *Cdk19* transcripts in cells carrying homozygous mutations in *Cdk19* (Fig. 2E). We did not detect a measurable change in the expression of *Cdk8* in *Cdk19* mutant cells. Similarly, no change of *Cdk19* expression in *Cdk8* mutant cells could be observed (Fig. 2E). These observations show that there is no reciprocal regulation at the transcriptional level between the two homologous kinases. Importantly, *Cdk8* and *Cdk19* double-deficient ESCs had an appearance similar to control ESCs, demonstrating that mediator kinases are dispensable for the self-renewal of pluripotent cells. This allowed us to analyse the effect of combined mediator kinase mutations on *Xist* function. In contrast to the *Cdk8* mutation, loss of *Cdk19* did not have a measurable effect on *Xist* function, as determined by our single cell survival assay (Fig. 2F). In addition, the combined mutations of *Cdk8* and *Cdk19* resembled the *Cdk8* mutation and did not further increase cell survival after *Xist* induction (Fig. 2F). These results strongly suggest that *Cdk19* does not contribute to *Xist* function in mouse ESCs and demonstrate a specific requirement of *Cdk8*.

### *Cdk8* acts downstream of *Xist* localization

We next analysed a potential effect of the *Cdk8* mutation on *Xist* expression and localization. *Xist* was detected in *Cdk8* mutant cells at a level comparable with control cells (Fig. 2H,I). We counted *Xist* clusters and pinpoint signals 24 h after *Xist* induction, and did not observe a statistically significant difference between wild-type, *Cdk8* mutant and complemented cells (Fig. S2C). Further quantification of total fluorescence of the *Xist* clusters confirmed that *Xist* clusters in wild-type and ΔCdk8 ESCs contained comparable amounts of *Xist* (Fig. S2D). The measurement of *Xist* transcript abundance by RT-PCR revealed a higher expression level in wild-type cells, compared with *Cdk8* mutant or complemented cells. However, there was no statistically significant difference between *Cdk8* mutant and complemented cells (Fig. S2E). The higher observed *Xist* expression in wild-type cells is probably due to the polyclonal nature of the parental HATX3 ESCs, which might have included some differentiated cells. To investigate a potential recruitment by *Xist*, we used ESCs expressing HA-tagged Cdk8 protein for immunofluorescence staining, together with Ezh2 antisera for identifying the X chromosome. HA-tagged Cdk8 showed a diffuse nuclear localization pattern without an enrichment over the Ezh2 cluster, whereas in cells that did not express HA-tagged Cdk8, no signal was observed (Fig. 2G). Taken together, our results show that mutation of *Cdk8* does not affect the expression and localization of *Xist*, which suggests that *Cdk8* acts downstream of *Xist* localization.

### *Cdk8* is required for the efficient recruitment of PRC2 by *Xist*

**Fig. 3.**
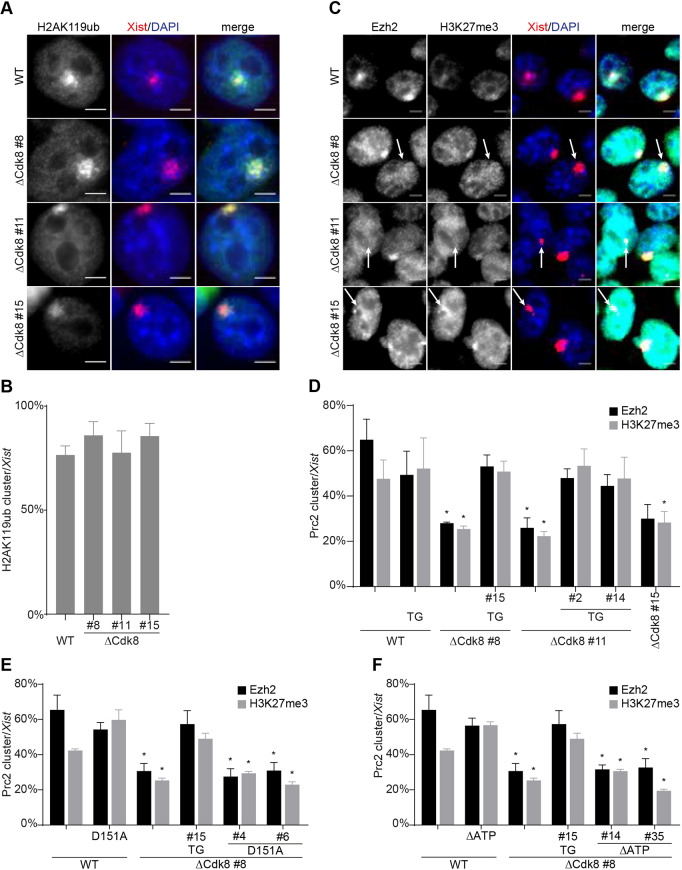
***Cdk8* is required for efficient PRC2 recruitment by *Xist*.** (A) Images of combined H2AK119ub immunofluorescence with *Xist* RNA FISH (red) for wild-type and ΔCdk8 cells. DAPI was used to stain DNA (blue). (B) Quantification of the percentage of *Xist* clusters with H2AK119ub foci after 24 h of *Xist* expression in ΔCdk8 and wild-type ESCs. Percentages are relative to counted *Xist* clusters; experiments were performed in triplicate; data are mean±s.d. (C) Combined immunofluorescence (Ezh2 and H3K27me3) with *Xist*-FISH (red) for wild-type and ΔCdk8 ESCs. White arrows indicate *Xist* clusters lacking PRC2 marks. DNA was stained with DAPI (blue). (D-F) Quantification of the percentage of *Xist* clusters with PRC2 (Ezh2 and H3K27me3) foci after 24 h of *Xist* expression in (D) ΔCdk8 ESCs and wild-type *Cdk8* transgene complemented ΔCdk8 ESCs, (E) D151A and (F) ΔATP mutant *Cdk8* transgene complemented ΔCdk8 ESCs. Percentages are relative to counted *Xist* clusters. Wild-type, ΔCdk8 #8 and ΔCdk8 #8 TG#15 samples are the same in E and F. The experiments were performed in triplicate. Data are mean±s.d.; asterisk indicates significant changes relative to WT (*P*<0.05). Scale bars: 5 μm.

To investigate a potential role for *Cdk8* in chromatin modifications of the Xi, we next measured the ability of *Xist* to recruit polycomb complex activity. We performed immunofluorescence staining with antisera specific for Ezh2, H3K27me3 and H2AK119ub in combination with *Xist* RNA fluorescent *in situ* hybridisation (FISH) after 24 h of *Xist* induction (Fig. 3, Fig. S3). We counted the number of foci relative to the number of *Xist* clusters (Fig. 3B-F). In ∼60% of nuclei, clear *Xist* clusters were detected using our combined staining technique, with little variation between different cell lines. Importantly, similar fractions of cells with *Xist* clusters were observed in cells mutant or wild-type for *Cdk8*, consistent with our earlier observation using *Xist* RNA FISH (Fig. S2C, Fig. S3A). The percentage of *Xist* clusters overlapping with H2AK119ub foci was comparable between *Cdk8* mutant and control cells (Fig. 3B). In wild-type cells, 40% of *Xist* clusters colocalised with H3K27me3 foci using our combined immunofluorescence RNA FISH staining technique (Fig. 3D-F). In cells lacking *Cdk8*, a significant reduction in H3K27me3 foci was observed, with ∼25-30% of *Xist* clusters colocalised with clear H3K27me3 foci (Fig. 3D). A similar pattern was observed for Ezh2 foci (Fig. 3D). Importantly, the reduction in Ezh2 and H3K27me3 clusters in *Cdk8* mutant cells was rescued by the expression of wild-type *Cdk8* cDNAs but not by catalytically inactive versions of Cdk8 (Fig. 3D-F, Fig. S3D-G, Fig. S4A). Therefore, the kinase activity of Cdk8 is required for the efficient recruitment of Ezh2 and PRC2 activity by *Xist*. To further investigate a general effect of *Cdk8* on polycomb histone modifications, we performed a western blot analysis using antisera specific for H3K27me3 and H2AK119ub (Fig. S3B). We observed comparable amounts of both histone modifications in control and ΔCdk8 ESCs, showing that *Cdk8* was specifically required for PRC2 recruitment by *Xist*.

### Mutation of *Cdk8* is embryonic lethal with a sex-specific phenotypic dimorphism

**Fig. 4.**
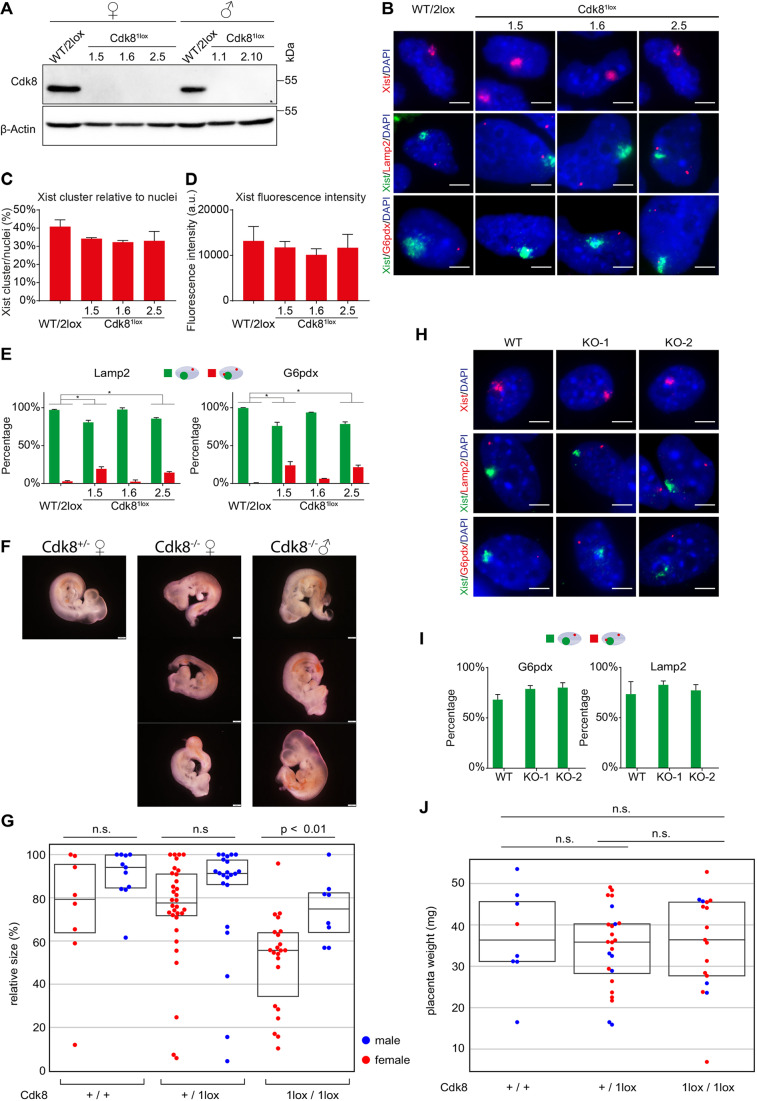
***Cdk8* contributes to the initiation of XCI in female mouse development.** (A) Western blot analysis of Cdk8 with β-Actin as loading control in ESCs established from embryos of crosses between *Cdk8*^WT/1lox^ Sox2-Cre^+/−^ males and *Cdk8*^2lox/2lox^ females. (B) RNA FISH expression analysis of *Xist* and X-linked genes *Lamp2* (middle row) or *G6pdx* (bottom row) in homozygous female *Cdk8*^1lox^ and control female ESCs after 96 h RA differentiation. Top row, *Xist* (red); middle and bottom rows, *Xist* (green) and X-linked genes (red), *Lamp2* (middle) and *G6pdx* (bottom) RNA FISH. DNA was stained with DAPI (blue). (C) Quantification of *Xist* RNA FISH analysis of *Cdk8*^1lox^ and control (WT/2LOX) female ESCs after 96 h RA differentiation. The percentage of *Xist* clusters is shown relative to counted nuclei (>100 counted). The experiments were performed in triplicate. Data are mean±s.d. a.u., arbitrary units. (D) Quantification of the fluorescence intensity of *Xist* clusters from RNA FISH (>100 clusters measured). The experiments were performed in triplicate. Data are mean±s.d. (E) Double FISH quantification of biallelic expression of the X-linked genes *Lamp2* and *G6pdx*, and overlap with an *Xist* cluster. Percentage (>100 *Xist* clusters analysed) of *Xist* clusters without detectable biallelic X-linked gene expression (green bars), and *Xist* clusters with overlapping X-linked gene expression (red bars) are shown. The experiment was performed in triplicate. Data are mean±s.d. Asterisk indicates a significant difference from the wild-type control (*P*<0.05). (F) Representative images of heterozygous control female (left), and homozygous female (middle) and male *Cdk8* mutant (right) embryos from one litter at E10.5. (G) Statistical analysis of embryo size as determined by image segmentation. The swarm plot shows the size normalized to largest size of the litter for each embryo with a box plot showing the mean and quartiles superimposed (n.s., not significant). (H,I) Female primary mouse embryonic fibroblasts derived from E10.5 *Cdk8*^1lox/1lox^ embryos. (H) RNA FISH expression analysis of *Xist* and X-linked genes *Lamp2* (middle) or *G6pdx* (bottom). Top, *Xist* RNA FISH (red); middle and bottom, *Xist* (green); and X-linked genes (red) RNA FISH. DNA was stained using DAPI (blue). (I) Quantification of RNA FISH analysis of biallelic X-linked gene expression in the presence of *Xist* clusters (as in B). The percentage of nuclei showing monoallelic expression of the X-linked genes *G6pdx* and *Lamp2* (green bars) in nuclei with *Xist* clusters are shown (>100 *Xist* clusters containing cells analysed). No cells with biallelic *Xist* expression were observed. The experiments were performed in triplicate. Data are mean±s.d. (J) Statistical analysis of placenta weights. Swarm plot showing the weight of individual placentae in mg for each genotype. Sex is indicated by the same colours as in G (n.s., not significant). Numbers above the lanes and panels in A and B, respectively, and on the *x*-axes in C, D and E indicate the clone numbers of independent ESC lines. Scale bars: 5 μm (B,H); 500 μm (F).

We next investigated whether there was a requirement for *Cdk8* in XCI. We established ESCs from blastocysts of a cross between females homozygous for a *Cdk8^2lox^* conditional mutation and heterozygous *Cdk8^1lox/+^* mutant males that also carry a Sox2-Cre transgene for epiblast-specific expression of Cre recombinase under the Sox2 regulatory region (Hayashi et al., 2002). We obtained two male and three female ESC lines that were homozygous for the *Cdk8*^1lox^ allele. Western blot analysis confirmed that Cdk8 protein was undetectable in these newly established ESCs (Fig. 4A). The potential for differentiation was further confirmed by analysis of *Pou5f1*, *Pax6* and *Gata6* expression before and after induction with retinoic acid (RA) for 4 days (Fig. S4B). Next, we investigated the expression of *Xist* and the X-linked genes *Lamp2* and *G6pdx* by RNA FISH after 4 days of differentiation in the presence of RA (Fig. 4B-E, Fig. S5A). The percentage of cells with *Xist* clusters and the fluorescence intensity of the *Xist* clusters were comparable between *Cdk8*-deficient and wild-type female ESCs (Fig. 4B-D). Furthermore, no difference in *Xist* abundance or *Pgk1* expression was observed by qRT-PCR (Fig. S5A, Fig. S4C). To assess silencing of *Lamp2* and *G6pdx*, we counted the percentage of cells that had FISH signals overlapping the *Xist* cluster, as well as cells that had a single FISH signal that did not overlap with *Xist* (Fig. 4E). In control cells, very few cells showed two signals for *Lamp2*, with one apparently originating from the Xi, as inferred from an overlap with *Xist*. Similarly, most cells displayed a single non-overlapping *G6pdx* signal. In contrast, in *Cdk8* mutant cells an increase in the number of cells with biallelic expression was observed for both X-linked genes, which was paralleled by an increase in the number of signals overlapping *Xist* (Fig. 4E). These data indicate that *Xist* did not silence *Lamp2* or *G6pdx* in a significant number of *Cdk8* mutant differentiating female ESCs, which is consistent with our earlier results showing that *Cdk8* is required for efficient gene repression by *Xist*.

Preliminary analysis of crosses between homozygous conditional *Cdk8^2lox^* mutant and heterozygous *Cdk8^1lox/+^* mutant Sox2-Cre mice indicated that the *Cdk8* mutation is lethal around embryonic day (E) 10.5 (Table S3, Fig. S4D,E). To assess a potential requirement of *Cdk8* for XCI in the embryo, we crossed mice that carried a heterozygous *Cdk8^1lox/+^* mutation. We obtained a total of 175 embryos, which included 43 homozygous *Cdk8^1lox/1lox^* mutant embryos (Table 1). The sex of these embryos was established by *Sry*- and *Zfy*-specific PCR to identify the Y chromosome. All female homozygous *Cdk8* mutant embryos that were obtained showed a more pronounced developmental delay and smaller size compared with males (Fig. 4F, Fig. S5B-D). We carried out the segmentation of microscopy images to determine the size of 103 embryos and analysed the statistical significance of the size difference between male and female embryos for all genotypes (Fig. 4G, Fig. S6, Table S4, Data S1; Wutz et al., 2020). The size difference between female and male homozygous *Cdk8* mutant embryos was statistically significant, whereas the sex-specific differences for other genotypes were not significant. The more severe phenotype of female *Cdk8* mutant embryos is consistent with a function of *Cdk8* in XCI.

**Table 1.**
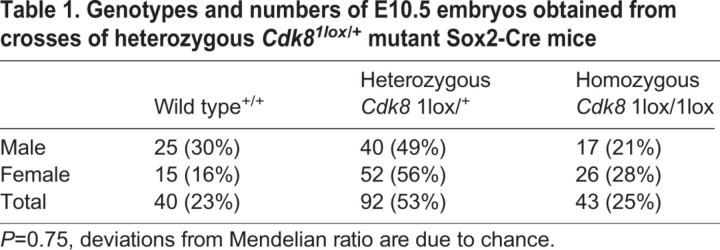
**Genotypes and numbers of E10.5 embryos obtained from crosses of heterozygous *Cdk8^1lox^*^/+^ mutant Sox2-Cre mice**

We established fibroblast cultures from wild-type and *Cdk8* mutant female embryos. We noted an impaired proliferation of *Cdk8* mutant fibroblast and could ultimately obtain two cultures (Fig. S5E) in which the expression of *Xist* (Fig. S5F) and X-linked genes could be examined. We detected no statistically significant difference in the percentage of cells with *Xist* clusters between wild-type and *Cdk8* mutant cells (Fig. 4H, Fig. S5G). In addition, the total fluorescence intensity of *Xist* clusters in *Cdk8* mutant cells was comparable with wild-type controls (Fig. S5H), showing that *Xist* was expressed and formed clusters in the absence of *Cdk8*. We then examined the expression of the X-linked genes *Lamp2* and *G6pdx* using RNA FISH (Fig. 4I). Both genes appeared efficiently silenced in wild-type and *Cdk8* mutant fibroblast cells. This finding indicated that the absence of *Cdk8* did not impair dosage compensation in somatic cells and suggests that *Cdk8* is a factor that contributes to the initiation of XCI.

To investigate whether a potential defect in the placenta could contribute to the phenotype of the *Cdk8* mutation, we determined the weight of placentae from E10.5 embryos of our heterozygous cross. We obtained weights for 51 placentae, which included 17 homozygous mutants (Fig. 4J, Fig. S7, Table S4, Data S1; Wutz et al., 2020). The weight difference between wild-type, homozygous and heterozygous, or male and female homozygous mutant placentae was not statistically significant, showing that placental weight was largely unaffected by the Cdk8 mutation. This observation suggests that the phenotype of the *Cdk8* mutation is caused by embryonic defects.

### Loss of *Cdk8* leads to the deregulation of gene expression associated with Notch signalling

To further investigate the lethality of the *Cdk8* mutation, we identified genes that are differentially expressed between wild-type and *Cdk8* mutant ΔCdk8 ESCs (Fig. S5I). Our RNAseq datasets revealed that 210 genes were significantly upregulated and 105 genes were downregulated in *Cdk8* mutant compared with wild-type control ESCs (Table S2). Among the upregulated genes were members of the *Zscan4* family that are associated with cleavage-stage transcriptional profiles and the two-cell-like state of mouse ESCs. Previous studies have shown that *Cdk8* regulates the Notch intracellular domain (Nicd). Consistent with this finding, we observed the upregulation of the Notch target gene *Hes1* in *Cdk8* mutant cells. We confirmed a 2.5-fold upregulation of *Hes1* in *Cdk8* mutant cells by RT-PCR (Fig. S5J). Investigation of the Nicd by western blot analysis indicated a higher level of the cleaved Nicd fragment in ΔCdk8 cells (Fig. S5K). Taken together, these findings indicate a deregulation of Notch signalling in the absence of *Cdk8*, which is consistent with the proposed role of *Cdk8* in the regulation of the Nicd. Although an increase in Notch activity was detectable, it did not affect the growth of *Cdk8* mutant ESCs, which is consistent with previous observations on overexpressing Nicd in ESCs (Lowell et al., 2006).

*Cdk8* has also been implicated in the activation of Stat1 and Stat3 by phosphorylation. Stat3 activation was of particular interest as it contributes to the stabilisation of pluripotent mouse ESCs (Ying et al., 2008). However, western blot analysis did not detect changes in Stat1 or in Stat3 phosphorylation (Fig. S5K). Importantly, we did not observe any changes in the expression of chromatin regulators, including polycomb group genes in *Cdk8* mutant cells. Overall, the mutation of *Cdk8* induced relatively few changes in the transcriptome of ESCs, suggesting it plays only a minor role in gene regulation. This observation indicates that the contribution of *Cdk8* to *Xist* function is not caused by transcriptome changes in ESCs.

## DISCUSSION

Our study implicates *Cdk8* as a new factor for X inactivation in mice. We found that *Cdk8* is required for efficient gene silencing by *Xist* and recruitment of PRC2 but is dispensable for *Xist* expression and localization. In the absence of *Cdk8*, PRC1 activity is recruited by *Xist*, which suggests there is a specific requirement for PRC2 recruitment. This is consistent with the current model of polycomb complex recruitment in X inactivation, which implies PRC1 activity as an initial signal for recruiting PRC2 (Almeida et al., 2017; Pintacuda et al., 2017).

The absence of *Cdk8* had a modest effect on gene expression in mouse ESCs. Notably, we did not detect changes in polycomb gene expression or in the expression of chromatin regulatory proteins among the top regulated genes. The effect of the *Cdk8* mutation was most dramatic on the repressive effect of *Xist* on X-linked genes. In the absence of *Cdk8*, X-linked genes remained, on average, expressed at half the level of the active X chromosome. In contrast, in control cells with an intact *Cdk8*, gene repression was almost complete. This effect on gene repression is consistent with an earlier observation of a repressive effect of *Cdk8* on super-enhancers in lymphoma cells (Pelish et al., 2015). This study showed that Cdk8 localizes to sites of mediator binding and acts to downregulate expression of associated genes. The remaining activity of *Xist* in *Cdk8* mutant cells is probably due to the activity of pathways that act in parallel.

We found that the paralogous kinase *Cdk19* is not required for *Xist* function. Considering the high level of sequence identity between the Cdk8 and Cdk19 proteins, this might appear surprising. Nonetheless, this observation is consistent with previous studies that have shown that Cdk8 and Cdk19 form distinct biochemical complexes that can act independently (Bourbon, 2008; Daniels et al., 2013). In our cell system, the *Cdk19* mutation did not have a measurable effect on *Xist* function and a combined mutation of *Cdk8* and *Cdk19* resembled the effect of the *Cdk8* mutation. Furthermore, we did not detect evidence of compensatory gene regulation between *Cdk8* and *Cdk19*, and neither mutation affected the expression of the respective other kinase gene. Therefore, we conclude that gene repression by *Xist* specifically requires Cdk8. Notably, the absence of both mediator kinases *Cdk8* and *Cdk19* does not impair ESC self-renewal. Homozygous *Cdk8 *and* Cdk19* mutant ESCs are a resource for future studies of mediator kinase function in signalling and gene expression.

Complementation experiments with mutant versions of *Cdk8* that are predicted to lack kinase activity, demonstrate that the catalytic activity of Cdk8 is required for *Xist* function. Interaction of mediator kinases with PRC2 subunits Ezh2 and Suz12, and phosphorylation of Ezh2, has been reported (Fukasawa et al., 2015), consistent with a direct role of Cdk8 kinase for PRC2 recruitment in X inactivation. We observed Cdk8 as a nuclear localized and chromatin-associated protein but we did not detect an enrichment over the X chromosome using HA-tagged Cdk8 expressing ESCs. Although the failure to detect an enrichment might be due to technical limitations, it is conceivable that Cdk8 kinase activity is locally activated on Xi by factors targeted by *Xist*. Cdk8 also does not contain a discernible RNA interaction motif. Notably, the cyclin-binding protein Ciz1 was previously observed on the Xi (Ridings-Figueroa et al., 2017). Ciz1 shows enrichment over the *Xist* domain at the initiation of XCI in ESCs but is not required until somatic cell fates are generated. It is enticing to speculate that Ciz1 could contribute to locally activate among other kinases, as well as Cdk8 (Andrau et al., 2006). However, this aspect would need further exploration in future studies.

Consistent with a requirement for X inactivation, we found that the *Cdk8* mutation causes a sex-specific dimorphic phenotype with a female-specific increased developmental delay at E10.5. Homozygous *Cdk8* mutant female embryos were smaller and had a more pronounced developmental delay compared with males. These observations are consistent with a defect in dosage compensation in female *Cdk8* mutant embryos. Although female embryo development is impaired more strongly than in males, lethality at E10.5 does not suggest a complete abrogation of dosage compensation. This observation is consistent with a partial defect of gene repression by *Xist* that is also gene specific in our ESC system. Our analysis further indicates the presence of an Xi in somatic cells in female *Cdk8* mutant embryos. These cells are probably selected in embryonic development as cells with dosage compensation defects are eliminated.

Our observation of a postimplantation lethality of *Cdk8* mutant embryos is inconsistent with an earlier study of a *Cdk8* gene trap mutation, which reported a developmental arrest in preimplantation embryos (Westerling et al., 2007). We did not detect Cdk8 protein in homozygous mutants of the *Cdk8*^1lox^ allele, which suggests a loss-of-function mutation. Statistical analysis shows that at E10.5 *Cdk8* mutant embryos are not under-represented and are observed at their expected Mendelian ratios. The discrepancy between our study and the earlier study can be reconciled by considering the different genetic backgrounds of the mouse strains and the differences in the structure of the *Cdk8* mutant alleles. In our hands, the *Cdk8* mutation is lethal around E10.5, with a more severe developmental delay in the female embryos that were recovered. From our placental weight measurements, we suggest that the *Cdk8* mutation predominantly affects the embryo and does not reduce the weight of the placenta at E10.5. We observed reduced embryo size and malformations with a striking defect in head development. This phenotype was associated with a delay or defect in neural tube closure. Taken together, our data demonstrate that *Cdk8* contributes to gene repression and PRC2 recruitment during the initiation of X inactivation, and is an essential gene for the post-implantation development of the mouse embryo.

## MATERIALS AND METHODS

### Cell lines

Diploidised HATX3 cells (Monfort et al., 2015), mutant derivatives and established ESC lines derived from mice were cultured as described previously (Wutz and Jaenisch, 2000). Briefly, cells were plated on gelatine-coated dishes containing high glucose Dulbecco's modified eagle medium (DMEM) (Life Technologies, 41965039) supplemented with 15% foetal bovine serum (Pan, P140402), 1% each of non-essential amino acids (Life Technologies, 11140035), sodium pyruvate (Life Technologies, 11360070) and L-glutamine (Life Technologies, 25030081), 8 μl/l β-mercaptoethanol (Sigma-Aldrich, M7522) and 1000 units/ml leukaemia inhibitory factor (LIF) (homemade). For maintenance culture, 3 μM Gsk3β inhibitor (Chir99021, Axon Medchem) and 1 μM Mek1/2 inhibitor (PD035901, Axon Medchem) were added (Ying et al., 2008). To induce *Xist* expression (Monfort et al., 2015) in HATX3 and mutant derivatives, 1 μg/ml doxycycline (Sigma-Aldrich, 324385) was administered. ESCs were differentiated for 96 h in the presence of 200 nM RA (Sigma-Aldrich, R2625). Primary mouse embryonic fibroblasts were cultured in a medium composed of high glucose DMEM (Life Technologies, 41965039) supplemented with 10% foetal bovine serum (Pan, P140402), 1% each of non-essential amino acids (Life Technologies, 11140035), sodium pyruvate (Life Technologies, 11360070) and L-glutamine (Life Technologies, 25030081).

*Cdk8* and *Cdk19* loss-of-function mutations were generated using a CRISPR/Cas9 strategy described previously (Monfort et al., 2015). Two guide RNAs targeting the region around the start codon were designed using the Massachusetts Institute of Technology algorithm (crispr.mit.edu) (Cdk8: gRNA1, 5′-ccggtccccaccgcggccct-3′ and gRNA2, 5′-aagttggtcgaggcacttac-3′; Cdk19: gRNA1, 5′-caccgtttcaaggcgaagctggcgg-3′ and gRNA2: 5′-caccgtaagagcgcgagcggggagt-3′) and inserted into PX330 vector (Addgene, 422300). Plasmids were sequenced for correct integration using a primer targeting the U6 promoter (5′-gactatcatatgcttaccgt-3′). The corresponding vectors, Cdk8 gRNA1/gRNA2 or Cdk19 gRNA1/gRNA2, were lipofected (Lipofectamine 3000, Thermo Fisher Scientific, L3000015) into HATX3 cells, together with tdTomato-N1 (Addgene, 54642), and after 48 h the cells were sorted for red fluorescence (MoFlo Astrios EQ, Beckman Coulter) and plated at limiting dilution in order to obtain clonal populations. Deletions were detected using PCR with genomic DNA (Cdk8: 5′-tctctcggaggagctaccggctgt-3′ and 5′-caaaactgagtgtcaccagccataggtttg-3′; Cdk19: 5′-ccaggttccaaaacaaggaa-3′ and 5′-acccctaaactccacctcca-3′) and validated by Sanger sequencing. Rescue and kinase-dead constructs were generated using the PiggyBac transposase system. Wild-type Cdk8 cDNA, reverse transcribed from RNA, was inserted into EcoRI-digested PB-EF1α-MCS-IRES-Neo vector (PB-EF1α-MCS-IRES-Neo cDNA cloning and expression vector, System Biosciences, PB533A-2) using a directional seamless cloning kit (In-Fusion HD Cloning Plus, Takara Bio, 638911) (primer: 5′-gcggccgatgactatgactttaaagtgaagctgagcag-3′ and 5′-ccgatttaaattcgaatttcagtaccgatgtgtctgatgtgagtac-3′). Additionally, DNA coding for an HA-Strep-tagII tag (primer: 5′-ctctagagctagcgaattatgtacccatacgatgttcccgac-3′ and 5′-gtcatagtcatcggccgctttttcgaac-3′) was cloned upstream of the Cdk8 cDNA. Site-directed mutagenesis to generate mutant Cdk8 cDNA vectors was achieved by an inverse PCR following the manufacturer's instructions (In-Fusion HD Cloning Plus, Takara Bio, 638911) (D151A: 5′-agggctttgaaacctgctaatattttagttatggg-3′ and 5′-gtttcaaagccctgtgcaacacc-3′; ΔATP: 5′-aaagactacgctttacaaatagaaggaactggaatttctatgtcgg-3′ and 5′-agttccttctatttgtaaagcgtagtctttatcgt-3′). Sequence-validated plasmids were lipofected into corresponding cells together with hyperactive PiggyBac transposase and a tdTomato fluorescent reporter in a ratio of 10:10:1, and sorted for tdTomato expression after 48 h. Independent clones were derived, selected for plasmid integration by the addition of 5 mg/ml G418 Sulphate (Life Technologies, 11811031), PCR screened for plasmid insertion (5′-gaccctgcttgctcaactct-3′ and 5′-tatagacaaacgcacaccg-3′) and validated by Sanger sequencing using the PCR screening primer. All cell lines tested negative for mycoplasma.

### Single cell survival assay

Single cells were sorted (MoFlo Astrios EQ, Beckman Coulter) into 96-well tissue culture plates containing ESC medium without Gsk3 and Mek1/2 inhibitors, which – within a plate – alternately contains 1 μg/ml Dox. The medium was changed after 5 days and emerging colonies were quantified after 12 to 14 days. The experiments were performed in technical duplicates and in biological triplicates.

### RNA extraction, cDNA synthesis and qPCR

RNA was extracted using the RNeasy Mini Kit (Qiagen, 74104) according to the manufacturer's protocol; including an on-column DNA digest using RNase-free DNase (Qiagen, 79254). RNA concentration was determined using a NanoDrop spectrophotometer. Equal amounts of RNA were deployed to reverse transcription, using the SuperScript IV reverse transcriptase kit (Thermo Fisher Scientific, 18090200). Oligo(dT)_15_ primer (Promega, C1101) was used to specifically reverse transcribe polyadenylated transcripts. qPCR experiments were performed in technical duplicates and biological triplicates using a 384-well format on a Roche 480 Lightcycler instrument using the SYBR Green method (KAPA SYBR FAST qPCR Kit, Kapa Biosystems, KK4611). Fold change expression was calculated by the ΔΔct method. *Gapdh* was used for normalisation.

### Primer sequences for gene expression analysis

The following primer sequences were used for gene expression analysis: Bex4, 5′-gataggcccaggagtgatg-3′, 5′-gggttcttcttcactttgtttg-3′; Cdk19, 5′-ggatctgtttgagtacgaaggg-3′, 5′-acaagccgacatagatattcctg-3′; Cdk8, 5′-agaggaaagatgggaaggac-3′, 5′-gctctcggagtaatgctatctc-3′; Gapdh, 5′-cgaaggtggaagagtgggag-3′, 5′-tgaagcaggcatctgaggg-3′; Hes1, 5′-caccggacaaaccaaagacg-3′, 5′-ggaatgccgggagctatctt-3′; Hmgn5, 5′-aaagaaaggctgcaggtg-3′, 5′-ggtttcaactccggtgtaaag-3′; Lef1, 5′-ccctgatgaaggaaagcatc-3′, 5′-gggtcgctgttcatattgg-3′; Mecp2: 5′-ccggggacctatgtatgatg-3′, 5′-aggaggtgtctcccaccttt-3′; Pdk3, 5′-gttcagagctggtacatgcag-3′, 5′-ggccattgtaggaacaacatc-3′; Pgk1, 5′-cccttcctggctatcttggg-3′, 5′-gatgtgccaatctccatgttgt-3′; Pls3, 5′-gcaggaatgaagcactgg-3′, 5′-cccatctcagcagaagctc-3′; Rrm2, 5′-cgccgagctggaaagtaaagcg-3′, 5′-tcgatgggaaagacaacgaagcg-3′; Xist, 5′-tgccatcctccctacctcagaa-3′, 5′-cctgacattgttttccccctaacaacc-3′; Pou5f1, 5′-caactcccgaggagtccca-3′, 5′- ctgggtgtaccccaaggtga-3′; Pax6, 5′-taacggagaagactcggatgaagc-3′, 5′-cgggcaaacacatctggataatgg-3′; Gata6, 5′gaagcgcgtgccttcatc-3′, 5′-gtagtggttgtggtgtgacagttg-3′; Atrx, 5′-cagtggatgatgacgacgac-3′, 5′-cccatcctcatcagagaaa-3′; Uba1, 5′-tttcctcctgaccagctc-3′, 5′-tttgggtccagaccagaa-3′; Armcx1, 5′-gggcagggtgcctgtatc-3′, 5′-ccttccctgcttcttggtttag-3′; and Huwe1, 5′-tgactacccccacaactg-3′, 5′-caccaacctttgctggag-3′.

### Western blot

Whole-cell lysates were prepared by resuspending cells in TNTE buffer [50 mM Tris (pH 7.5), 150 mM NaCl, 0.5% Triton X-100 and 1 mM EDTA] supplemented with 1 mM phenylmethylsulfonyl fluoride (PMSF), 10 mM MgCl_2_, 5 mM CaCl_2_ and 3000 U/ml DNase I, and incubation on a rotating wheel for 30 min at 4°C followed by 20 min centrifugation at 18,000 ***g***. Cellular fractions were prepared as described previously (Graumann et al., 2008) with minor modifications. Briefly, collected cells were incubated for 10 min in ice-cold buffer containing 10 mM HEPES (pH 7.9), 1.5 mM MgCl_2_ and 0.2% Igepal-C630, supplemented with 1 mM PMSF. The suspension was homogenised using a glass douncer and a loose pestle. After centrifugation for 15 min at 400 ***g***, the supernatant containing cytoplasmic proteins was collected and stored on ice. The pellet was washed in ice-cold PBS and resuspended in buffer, containing 420 mM NaCl, 20 mM HEPES (pH 7.9), 20% glycerol, 2 mM MgCl_2_, 0.2 mM EDTA, supplemented with 1 mM PMSF, and incubated on ice for 60 min. After centrifugation for 15 min at 18,000 ***g***, the nucleoplasmic protein-containing supernatant was collected and stored on ice. The pellet was washed in ice-cold PBS and resuspended in PBS supplemented with 600 mM NaCl, 1% Igepal-C630, 10 mM MgCl_2_, 5 mM CaCl_2_ and 3000 U/ml DNase I, and incubated on a shaker at 10°C at 600 rpm for 30 min. After centrifugation for 15 min at 18,000 ***g*,** the chromatin-bound protein containing supernatant was collected and stored on ice. Histones were extracted by pre-extraction using 1 ml 0.5% Triton-X100 in PBS+2 mM PMSF per 10^7^ cells followed by 10 min centrifugation at 400 ***g*** at 4°C. Resulting pellets were incubated in 0.2 M HCl (1 ml/4×10^7^ cells) overnight at 4°C followed by 10 min centrifugation at 400 ***g*** at 4°C. Protein concentrations were determined using the Bio-Rad DC Protein Assay (DC Protein Assay Kit II, Bio-Rad, 5000112). Equal protein concentrations for total cell lysates and equal volumes for cellular fractions were subjected to SDS-PAGE, and blotted on polyvinylidene difluoride membranes. The membranes were blocked in TBS+0.1% Tween 20 (TBST)+5% non-fat dried milk. Membrane washes were performed in TBST and antibody incubations were performed in TBST+5% non-fat dry milk. Blots were developed using the enhanced chemiluminiscence detection method on a Bio-Rad ChemiDoc system. Clarity Western ECL substrate (Bio-Rad, 1705060) was used for abundant proteins and SuperSignal West Femto Maximum Sensitivity Substrate (Thermo Fisher Scientific, 34095) was applied for proteins expressed at low levels.

Primary antibodies used for immunoblotting were as follows: goat anti-Cdk8 (Santa Cruz Biotechnology, SC-1521, dilution 1:400), anti-β-Actin-HRP (Sigma-Aldrich, A3854, dilution 1:20,000), rabbit anti-Notch1 (Cell Signaling Technology, 3608S, dilution 1:500), rabbit anti-phospho-Stat1(S727) (Cell Signaling Technology, 9177S, dilution: 1:200), mouse anti-Stat1 (BD Biosciences, 610186, dilution: 1:1000), rabbit anti-phospho-Stat3(S727) (Cell Signaling Technology, 9134T, dilution: 1:500), mouse anti-Stat3 (Cell Signaling Technology, 9139T, dilution: 1:1000), rabbit anti-Oct4 (Santa Cruz Biotechnology, sc-9081, dilution: 1:250), mouse anti-α-Tubulin (GenScript, A01410-40, dilution: 1:30,000), mouse anti-H3 (Cell Signaling Technology, 3638T, dilution 1:1000) and rabbit anti-H2AK119ub (Cell Signaling Technology, 8240S, dilution 1:2000). Secondary antibodies, purchased from Jackson ImmunoResearch and used at a dilution of 1:20,000, were as follows: peroxidase AffiniPure bovine anti-goat IgG (H+L) (805-035-180), peroxidase AffiniPure donkey anti-mouse IgG (H+L) (715-035-180) and peroxidase AffiniPure donkey anti-rabbit IgG (H+L) (711-035-152).

### RNA FISH

RNA FISH was performed as described previously (Wutz and Jaenisch, 2000). Cells were seeded on laminin- (Sigma-Aldrich, L2020-1MG) coated (5 μg/ml in PBS) Roboz slides (CellPoint Scientific) in appropriate medium. Cells were rinsed in PBS, washed in cytoskeletal (CSK) buffer [10 mM PIPES (pH 6.8), 100 mM NaCl, 300 mM sucrose and 3 mM MgCl_2_] and proteins were extracted by incubation in CSK buffer, supplemented with 0.5% Triton X-100, for 7 min. After one wash in CSK buffer, cells were fixed in 4% paraformaldehyde in PBS for 10 min at room temperature. Slides were dehydrated in an ethanol series of 70%, 80%, 95% and 100%. FISH probe was applied to the cells and incubated overnight at 37°C in a light-protected humidified chamber. Slides were washed three times each for 5 min at 39°C with 50% formamide/2× SSC [300 mM NaCl, 30 mM sodium citrate (pH 7.0)] and 2× SSC, respectively, followed by one wash with 1× SSC. Cellular DNA was stained with DAPI and slides were mounted with mounting medium (Vectashield H-1000, Vectorlabs, H-1000). *Xist* FISH probes were prepared as described previously (Karen and Wutz, 2007) using the random primer labelling technique (Prime-It II Random Primer Labelling Kit, Stratagene, 300385) and Cy3-dCTP using a Xist cDNA template [blueprint vector containing Xist cDNA, pBP5.6 (Wutz and Jaenisch, 2000)]. For double FISH experiments, *Xist* probes were prepared using Fluorescein-High Prime kit (Sigma-Aldrich, 11585622910) and X-linked gene probes were prepared using the random primer labelling technique and Cy3-dCTP using genomic bacterial artificial chromosome templates (Lamp2: RP24173A8, G6pdx: RP2313D21).

### Combined immunofluorescence and RNA-FISH

This experiment was performed as described previously (Chaumeil et al., 2008) with minor modifications. Cells grown as mentioned for RNA FISH, were rinsed with ice-cold PBS and fixed for 10 min in 4% paraformaldehyde in PBS at room temperature. After two washes in PBS, cells were permeabilised in 0.1% sodium citrate/0.5% Triton X-100 in PBS supplemented with 2 mM ribonuleoside vanadyl complex (RVC) (NEB, S1402S) for 10 min on ice. Slides were washed two times in ice-cold PBS+0.1% Tween 20 (PBS-T) and blocked for 45 min at room temperature in 2.5% BSA in PBS-T supplemented with 2 mM RVC. Primary antibodies [mouse anti-Ezh2 (AC22) (dilution 1:100, Cell Signaling Technology, 3147); rabbit anti-H3K27me3 (dilution 1:200, Active Motif, 39155); rabbit anti-HA (C29F4) (dilution 1:1000, Cell Signaling Technology, 3724); rabbit anti-H2AK119ub (dilution 1:500, Cell Signaling Technology, 8240)] diluted in blocking solution supplemented with 2 mM RVC and 1 U/μl RiboLock RNase inhibitor (Thermo Fisher Scientific, EO0381), were added to the cells for 60 min at room temperature. Following three washes with PBS-T, fluorophore-labelled secondary antibodies [Alexa Fluor 488 AffiniPure donkey α-mouse IgG (H+L), Jackson ImmunoResearch, 715-545-150; and Alexa Fluor 647 AffiniPure Donkey α-Mouse IgG(H+L), Jackson ImmunoResearch, 715-605-150], in blocking solution/RVC/RNase inhibitor, were applied at a dilution of 1:1000 and incubated for 60 min at room temperature in the dark. Slides were washed twice with PBS-T and once with PBS. Slides were post-fixed for 10 min with 4% paraformaldehyde in PBS at room temperature, washed once in PBS followed by a wash in 2× SSC. After air drying of the samples, the FISH protocol applied from the point of adding the probe to the slides.

### Microscopy

Samples were analysed with a Zeiss Axio Observer Z.1 fluorescence microscope equipped with a Hamamatsu OrcaFlash 4.0 camera and a Plan Apochromat 100×/1.46 oil DIC objective. Images were processed using Zeiss Zen Pro 2.0 software and figures were prepared using ImageJ/Fiji and Adobe Photoshop to crop pictures and adjust brightness and contrast. Fluorescence intensity measurements were taken by measuring the fluorescence of a defined area with a fixed exposure time, and intensity was calculated by subtracting background fluorescence. Experiments were performed in triplicates and more than 100 measurements or counts were taken for each experiment. Embryo images were acquired using an Olympus MVX10 Stereo-Zoom microscope equipped with an Olympus DP73 camera using the cellSens software. Image segmentation was performed using a custom Python script (Data S1; Wutz et al., 2020) from the scikit-image package. Statistical analysis was performed using a custom Python script based on the scikit-stats package (see supplementary Materials and Methods).

### Mouse experiments

All *in vivo* experiments were performed under the licence ZH152/17 in accordance with the standards and regulations of the *Kantonale Ethikkommission Zürich*. Breeding, maintenance, timed matings and plug checks were carried out at the ETH Phenomics Center Mouse Facility. B6.CDK8tm1(fl/fl)Eucomm mice were a kind gift from Professor Markus Stoffel (ETH Zurich, Switzerland) and B6.Cg-Tg(Sox2-cre)1Amc/J (Hayashi et al., 2002) mice were kindly provided by Professor Jennifer Nichols (University of Cambridge, UK). Embryos were collected in accordance with Institute of Molecular Health Sciences regulations. Pictures were taken at the same magnification using an Olympus MVX10 stereo microscope mounted with an Olympus DP73 camera. Pictures were prepared using Adobe Photoshop to crop and adjust brightness and contrast. ESCs were derived in accordance with published procedures (Nichols and Jones, 2017). Briefly, eight-cell-stage embryos were flushed from oviducts at 2.5 dpc and incubated in KSOM medium (Millipore, MR-020P-5D), supplemented with 3 μM Gsk3β inhibitor (Chir99021, Axon Medchem) and 1 μM Mek1/2 inhibitor (PD035901, Axon Medchem) overnight, at 37°C/5% CO_2_. The next day, the medium was replaced with N2B27 (Takara Bio, Y400002), supplemented with Chir99021+PD0335901, and incubated for another 2 days. Inner cell masses were isolated by immunosurgery consisting of incubation in N2B27 with 20% rabbit α-mouse serum (Sigma-Aldrich M5774) for 1 h at 37°C, 5% CO_2_, followed by incubation in N2B27 with 20% guinea pig serum (Sigma-Aldrich, G9774) for 10 min. Isolated epiblasts were placed in tissue culture plates containing N2B27 supplemented with Chir99021, PD035901 and LIF. Primary mouse embryonic fibroblasts were derived from E10.5 embryos by pushing the embryos through a 27-gauge needle. Expanded cell lines were genotyped using the following primers: Cdk8 deletion, 5′-cttccctcttcccagaggac-3′, 5′-caaccccttttgaggttgaa-3′; Xist: 5′-gtagatatggctgttgtca-3′, 5′-ctccatccaagttctttctg-3′; Sry: 5′-tcttaaactctgaagaagagac-3′, 5′-gtcttgcctgtatgtgatgg-3′; and Zfy: 5′-aagataagcttacataatcacatgga-3′, 5′-cctatgaaatcctttgctgcacatgt-3′. Embryos were genotyped for Cdk8 deletion using the following primers: 5′-ggtgctggaggattaagtgc-3′, 5′-cacagaggacagcacagagc-3′.

### Transcriptomic analysis

RNA was extracted as described above and sequenced by the Functional Genomics Center Zürich. Polyadenylated RNA was enriched using Oligo(dT) beads and libraries were prepared using an Illumina TruSeq Stranded mRNA kit. Libraries were sequenced on an Illumina HiSeq 4000 machine, creating 125 base single-end reads. CLC Genomics Workbench 11 (Qiagen), licensed by ETH, was used for analysis. Adapters were not trimmed and the reads were aligned to the Ensembl reference genome (GRCm38.94). Reads were normalised using the transcripts per kilobase million method. Statistically significant differentially expressed genes were obtained by comparing the data set of interest with a control group, meaning treated against non-treated for differentially regulated X-linked genes or ΔCdk8 non-treated against wild type non-treated for general transcriptional changes using a Wald test. Confounding factors were not taken into consideration. The threshold for significance was set to an absolute fold change greater than two and a false discovery rate *P*-value less than 0.01.

### Statistics

Paired parametric two-tailed *t*-tests were conducted to determine significance with a *P*-value threshold less than 0.05. Calculations were performed using Prism GraphPad Version 7.

## Supplementary Material

Supplementary information
