# Supplementary material for: Cdk8 is required for establishment of H3K27me3 and gene repression by Xist and mouse development
Source: Development. 2020 Jun 11;147(11):dev175141. doi: 10.1242/dev.175141 (PMC7295591; doi:10.1242/dev.175141)
Supplement: Supplementary information [file develop-147-175141-s1.pdf]

Table S1. Top 100 repressed X-linked genes in WT upon *Xist* induction. Sorted column-wise for descending fold change (FC) in the WT samples. FDR p-value: false discovery rate corrected p-value; WT: data for WT samples; ΔCdk8: corresponding FC and FDR p-value for ΔCdk8 samples; Red: genes significantly downregulated in WT and ΔCdk8 samples.

Gene	WT FC	WT FDR p-value	ΔCdk8 FC	ΔCdk8 FDR p-value
Slc16a2	-132.400	0.000	-12.710	0.100
Armcx6	-75.570	0.000	-4.820	0.470
Gabra3	-53.610	0.000	-10.340	1.000
Armcx2	-42.560	0.000	-5.950	0.000
Tmem28	-41.450	0.000	-5.230	0.290
Usp51	-29.950	0.000	-4.270	1.000
Heph	-28.340	0.000	-4.020	1.000
Zcchc18	-27.400	0.000	-2.700	1.000
Apln	-26.320	0.004	-5.700	1.000
Armcx3	-24.170	0.000	-5.850	0.050
Magee2	-22.830	0.000	-3.560	1.000
Nhsl2	-22.780	0.000	-8.170	0.020
Rab33a	-22.680	0.000	-2.910	1.000
Tceal1	-21.440	0.000	-2.180	1.000
Smarca1	-20.190	0.000	-3.840	0.580
Tceal5	-19.660	0.001	-2.330	1.000
405ERik	-19.640	0.000	-6.440	0.220
Cysltr1	-18.240	0.000	-2.140	1.000
Nap1l2	-18.230	0.000	-4.780	1.000
Fndc3c1	-17.930	0.000	-3.070	1.000
Nlgn3	-17.740	0.000	-2.810	1.000
Fthl17a	-17.330	0.010	-2.150	1.000
Phka1	-17.250	0.000	-3.650	0.050
Nxf3	-16.880	0.000	-2.680	1.000
Foxr2	-16.330	0.000	-5.040	0.860
Gpr173	-16.300	0.000	-2.340	1.000
Usp26	-15.880	0.000	-4.090	0.130
Tsc22d3	-15.020	0.000	-2.420	1.000
Uprt	-14.650	0.000	-4.310	0.640
Slitrk2	-14.630	0.000	-2.730	1.000
Dmrt1c2	-14.600	0.000	-2.740	1.000
Armcx4	-14.280	0.000	-3.710	0.001
Stk26	-14.000	0.000	-3.470	0.220
Igsf1	-13.950	0.000	-2.860	1.000
Sox3	-13.920	0.000	-1.420	1.000
Gk	-13.880	0.000	-2.710	0.930
Hdx	-13.870	0.000	-2.540	1.000
Armcx1	-13.750	0.000	-4.180	0.001
Gabre	-13.730	0.003	-2.930	1.000
Rragb	-13.090	0.000	-4.160	0.050
Taf9b	-12.930	0.000	-2.260	1.000
Gpc3	-12.600	0.000	-2.580	0.410
Tex11	-12.360	0.000	-2.220	0.470
Rlim	-12.140	0.000	-3.550	0.020
Zcchc12	-12.130	0.000	-2.120	1.000
Fgf13	-11.950	0.000	-2.270	1.000
Pls3	-11.830	0.000	-2.750	0.220
Eda	-11.690	0.000	-3.690	0.860
Ophn1	-11.630	0.000	-2.720	1.000
Tsx	-11.510	0.000	-3.700	1.000
Sh3bgrl	-11.510	0.000	-2.330	1.000
Zxdb	-11.440	0.000	-2.430	1.000
Ccdc160	-11.080	0.000	-3.420	0.380
Tro	-10.940	0.000	-3.190	0.010
Gm364	-10.480	0.002	-2.890	1.000
Lonrf3	-10.410	0.000	-2.080	1.000
Kctd12b	-10.340	0.000	-2.170	1.000
Fmr1nb	-10.280	0.000	-2.160	1.000
Cited1	-10.010	0.005	-3.000	1.000
Prrg3	-9.990	0.000	-2.680	1.000
Lpar4	-9.920	0.000	-4.190	1.000
Spin4	-9.840	0.000	-1.930	1.000
Pdk3	-9.700	0.000	-2.240	0.320
Irs4	-9.300	0.003	-2.300	1.000
Awat2	-9.290	0.002	-2.700	1.000
Ldoc1	-9.100	0.000	-3.110	1.000
Fam199x	-9.000	0.000	-1.960	1.000
Acsl4	-8.930	0.000	-1.820	1.000
Rab39b	-8.890	0.000	-2.550	1.000
Bex4	-8.680	0.000	-2.030	0.440
Zdhhc15	-8.560	0.000	-2.160	1.000
Egfl6	-8.540	0.000	-2.020	1.000
Msn	-8.320	0.000	-1.920	0.580
Pja1	-8.310	0.000	-2.940	0.020
Tmem164	-8.130	0.000	-1.720	1.000
Smim1l2a	-8.090	0.000	-2.200	1.000
Tceal8	-8.040	0.000	-2.090	0.790
Pgk1	-7.990	0.000	-3.090	0.000
Amer1	-7.950	0.000	-3.230	0.080
Mum1l1	-7.690	0.000	-2.650	1.000
Maged2	-7.690	0.000	-3.780	0.020
Zfp280c	-7.660	0.000	-2.400	0.580
Rtl8a	-7.570	0.001	-2.150	1.000
Maged1	-7.570	0.000	-2.740	0.001
Fhl1	-7.550	0.000	-2.110	1.000
Klf8	-7.480	0.000	-2.560	0.430
Bex1	-7.460	0.000	-1.800	1.000
Dlg3	-7.400	0.000	-1.900	1.000
Tspan6	-7.350	0.000	-2.020	1.000
Dmd	-7.260	0.000	-2.950	0.220
Amot	-7.250	0.000	-1.960	1.000
Nxt2	-7.130	0.000	-2.740	0.580
Ripply1	-7.000	0.002	-7.090	0.580
Pcyt1b	-6.860	0.000	-2.980	0.030
Cd99l2	-6.730	0.000	-2.430	1.000
Tceal9	-6.680	0.000	-1.900	1.000
Map7d3	-6.660	0.000	-3.640	1.000
Arxes1	-6.510	0.000	-2.310	1.000
Morf4l2	-6.510	0.000	-2.610	0.001
Tspan7	-6.440	0.000	-1.970	1.000

Development • Supplementary information

Table S2. Top 50 differentially expressed genes in ΔCdk8 compared to WT ESCs. Genes up regulated (left column) and down regulated (right column) in ΔCdk8 ESCs were sorted for descending fold change (FC). FDR p-value: false discovery rate corrected p-value

Activated Genes in ΔCdk8 over WT			Repressed Genes in ΔCdk8 over WT		
**Gene**	**FC**	**FDR p-value**	**Gene**	**FC**	**FDR p-value**
Gal3st2c	95.35	0.005	Gm28635	-65.25	0.001
S100a4	48.69	0.001	Spon1	-38.88	0.000
Ly6a	43.26	0.000	Dlx6	-27.67	0.005
Ankrd1	38.61	0.006	En1	-26.92	0.000
Nlrp4a	37.98	0.000	Mest	-24.03	0.000
Gm5662	37.73	0.001	Slitrk2	-20.07	0.000
Msln	35.63	0.000	Calcr	-19.8	0.000
Chit1	34.97	0.000	Sema6a	-16.51	0.000
Prnd	34.22	0.006	Alox5	-16.34	0.001
17000Rik	30.01	0.000	Enpp2	-12.44	0.000
Kng2	27.88	0.001	Cdh9	-11.58	0.000
Zscan4-ps	27.8	0.000	Dgki	-11.53	0.000
Zscan4d	22.43	0.006	Gabra3	-10.5	0.001
Cdh23	20.75	0.002	Pim2	-10.34	0.000
Zscan4e	20.11	0.006	Ror1	-9.52	0.000
Gm5039	17.79	0.008	Peg3	-9.25	0.000
Aox3	16.89	0.000	Has2	-9.23	0.000
Smc1b	14.65	0.000	Fgf5	-8.84	0.002
Ott	14.3	0.000	Cxcl12	-8.69	0.000
Zscan4c	14.24	0.010	Ar	-8.27	0.006
Inhbb	14.08	0.000	Zfp608	-7.79	0.000
Xlr3a	14.04	0.003	Msx1	-7.62	0.004
Crtac1	13.99	0.000	Lef1	-7.57	0.000
Cdh13	13.84	0.000	Cdkn1a	-7.44	0.000
Bgn	13.43	0.002	Ndnf	-7.38	0.005
Rnase1	13.25	0.005	Tcte2	-6.33	0.000
Loxl4	13.09	0.000	Cnr1	-6.16	0.006
Sh2d4b	12.83	0.003	Vrtn	-5.82	0.001
Nipal1	12.79	0.006	Myb	-5.78	0.000
Slc13a5	12.75	0.000	Erdr1	-5.22	0.001
Phf11b	12.59	0.008	Aff3	-5.2	0.000
Mmrn2	12.52	0.000	Prrt3	-5.11	0.008
Krtdap	12.46	0.001	Lpar4	-5.08	0.001
Adh7	12.26	0.009	Gm45062	-5.03	0.000
Gm15097	11.83	0.002	Zic2	-4.9	0.000
Emp1	11.83	0.000	Id3	-4.71	0.000
Tex13b	11.55	0.001	Ccng1	-4.66	0.001
Hck	11.45	0.001	Plk2	-4.59	0.001
Ddr2	11.18	0.004	Pou3f1	-4.58	0.000
Il6ra	11.02	0.000	Sall2	-4.38	0.003
Fam25c	10.95	0.000	Tmem267	-3.99	0.009
C1qtnf9	10.06	0.000	Car14	-3.59	0.004
Slc44a3	10.03	0.002	Otx2	-3.55	0.000
Hspb2	10.01	0.006	Lrp2	-3.35	0.000
Pdgfrb	9.87	0.005	Ina	-3.3	0.000
Ly6g6e	9.58	0.000	Ppp1r10	-3.24	0.004
Snrpn	9.4	0.000	Sox4	-3.23	0.000
Cryab	8.64	0.001	Cd24a	-3.22	0.000
Anxa3	8.4	0.000	Zhx1	-3.13	0.008
Synpo2l	7.99	0.001	Foxp1	-3.11	0.000

Development • Supplementary information

Table S3. Complete penetrance of lethality of the homozygous *Cdk8* mutation in mice. Genotypes and number of viable offspring obtained from conditional and heterozygous crosses of the *Cdk8* mutation

Cdk8$^{2lox/2lox}$ females x *Cdk8*$^{1lox/+}$ Sox2-Cre males (epiblast specific *Cdk8* mutation)

	Cdk8$^{2lox/+}$		*Cdk8*$^{1lox/+}$ Sox2-Cre		*Cdk8*$^{1lox/2lox}$		*Cdk8*$^{1lox/1lox}$ Sox2-Cre		total		
	M	F	M	F	M	F	M	F	M	F	all
weaning	6	7	8	8	8	10	**0**	**0**	22	25	47

Cdk8$^{1lox/+}$ x *Cdk8*$^{1lox/+}$ (heterozygous *Cdk8* cross)

	Cdk8$^{+/+}$		*Cdk8*$^{1lox/+}$		*Cdk8*$^{1lox/1lox}$		total		
	M	F	M	F	M	F	M	F	all
weaning	2	6	8	8	**0**	**0**	10	14	24

Table S4. Analysis of *Cdk8* mutant embryo size and placental weight. An excel workbook containing embryo numbers, embryos sizes determined by image segmentation, and placental weights used for statistical analysis in Table 1, and Fig. 4G and 4J. The p-value for deviation from Mendelian expected ratios for Cdk8 embryos was calculated using a Chi-Square test. p-values for embryo size and placental weight differences were calculated using a two-sided t-test and are included in separate excel sheets within the workbook.

Click here to Download Table S4

Development • Supplementary information

Supplementary Materials and Methods

Data S1. Python scripts for image and statistical analysis of *Cdk8* mutant embryos and placentae. EmbryoImage.py is a Python script that performs automated image segmentation and calculates the size of embryos in pixel from microscopy images. The script uses the scikit-image module. Embryo images, segmentation results, and sizes are included in the Dryad Digital Repository (Wutz et al., 2020). Embryos sizes were normalized to the size of the largest embryo per litter and plots (Fig. 4G) and p-values were generated using the plot_Cdk8_embryo_sizes.py script. The script Cdk8_plot_placenta_weight.py was used to plot placental weights of Cdk8 mutant placentae (Fig. 4J) and generate p-values. An excel workbook with the measurements and all p-values for embryo sizes and placental weights is included in Table S4.

Development • Supplementary information

Figure S1

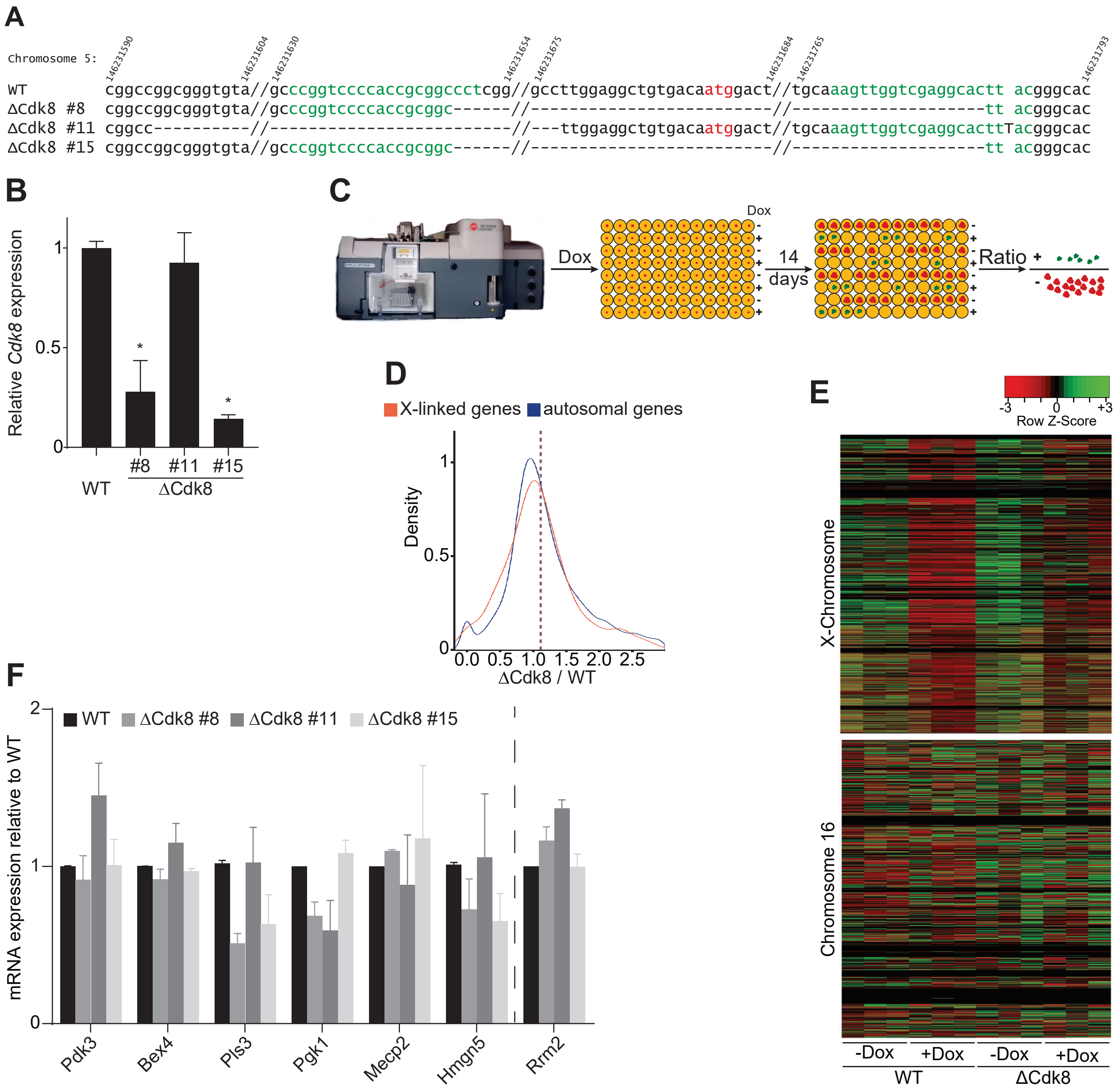

Development • Supplementary information

Figure S1. Loss of *Cdk8* results in reduced X-linked gene silencing by *Xist*. (A) Sequencing data of the deletion fragment in ΔCdk8 #8, #11 and #15 cells. Numbers above indicate genomic location, green characters indicate gRNA binding sites; red characters indicate start codon, capital character indicates single base insertion. **(B)** qRT-PCR validation of CRISPR/Cas9 engineered ΔCdk8 HATX3 cells using primers in *Cdk8* exon 2 and 3. Expression levels are normalised to *Gapdh* and relative to WT; experiments performed in triplicates; error bars represent SD; asterisk indicates significant changes relative to WT (*P*-value<0.05). **(C)** Schematic representation of single cell survival assay to assess *Xist* function. Single cells were sorted into 96-well plates with ESC medium containing Doxycycline to induce *Xist* expression (+) or without Doxycycline (-). Colonies in the presence of Dox (+) were quantified relative to control conditions (-). **(D)** RNAseq data representation of gene expression ratio of ΔCdk8 cells relative to WT in untreated conditions. The ratio of X-linked (red curve) and autosomal (blue curve) gene expression is shown; dashed lines indicate median values. **(E)** Heatmap of TPM normalised RNAseq expression values without clustering for WT and ΔCdk8 replicates after 48 hours of *Xist* expression and in control conditions for the X-chromosome (top), and chromosome 16 (bottom, as control) are shown. Relative expression is color coded in green (high expression) to red (low expression). Heatmaps were generated by www.heatmapper.ca. **(F)** Expression levels of X-linked genes in ΔCdk8 cells compared with WT cells in untreated conditions; *Rrm2* serves as autosomal control; normalised to *Gapdh* and relative to WT; experiments performed in triplicates; error bars represent SD.

Development • Supplementary information

Figure S2

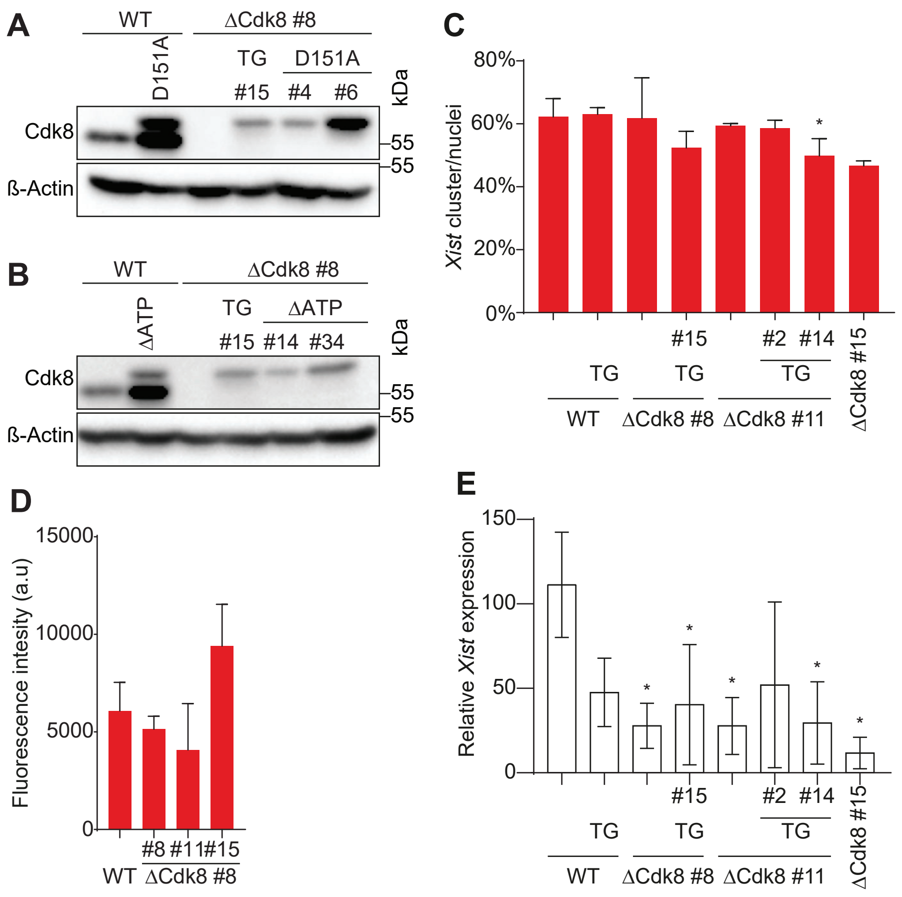

Development • Supplementary information

Figure S2. *Cdk8* acts downstream of *Xist*. (A-B) Immunoblot confirming expression of **(A)** Cdk8 D151A and **(B)** ΔATP mutant transgene in ΔCdk8 #8 and WT ESCs, respectively; β-Actin: loading control. **(C)** Quantification of *Xist* clusters from RNA FISH after 24 hours of *Xist* expression in ΔCdk8 and WT *Cdk8* transgene complemented ΔCdk8 ESCs. Percentage relative to counted nuclei are shown (>100 counted); experiment performed in triplicates; asterisk indicates significant change relative to WT (*P*-value<0.05). **(D)** Quantification of fluorescence intensity of *Xist* clusters from RNA FISH (>100 cluster measured); experiments performed in triplicates; error bars represent SD. **(E)** qRT-PCR analysis of *Xist* expression in ΔCdk8 and WT Cdk8 transgene complemented ΔCdk8 cells; expression levels normalised to *Gapdh* and relative to uninduced conditions; asterisk indicates significant changes relative to WT (*P*-value<0.05).

Development • Supplementary information

Figure S3

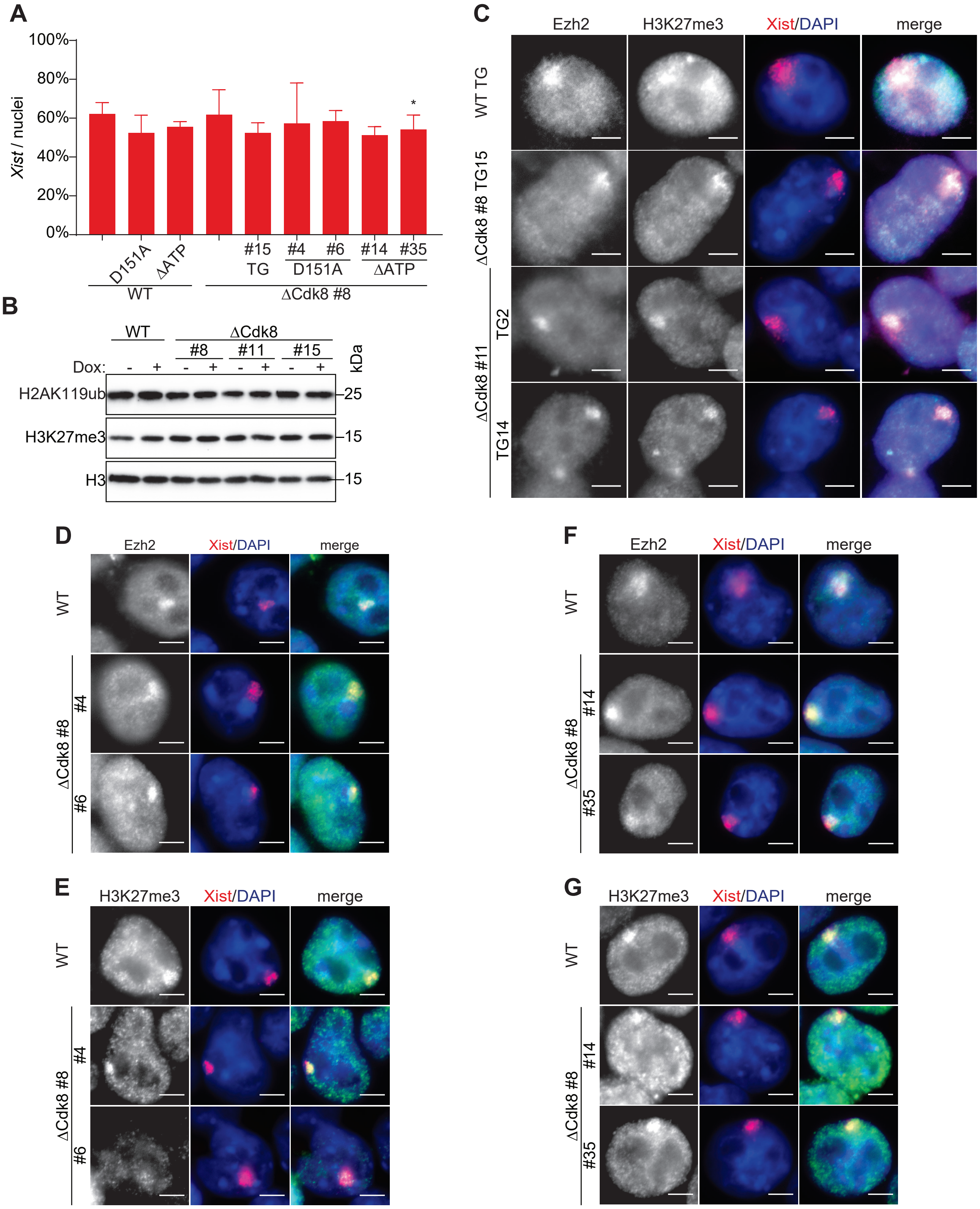

Development • Supplementary information

Figure S3. *Cdk8* is required downstream of *Xist* for recruitment of PRC2. (A) Quantification of *Xist* clusters from RNA FISH after 24 hours of *Xist* expression in D151A and ΔATP mutant transgene complemented ΔCdk8 cells. Percentage relative to counted nuclei (>100 counted); experiments performed in triplicates; asterisk indicates significant change relative to WT (*P*-value<0.05). **(B)** Immunoblot showing H2AK119ub (top) and H3K27me3 (middle) in ΔCdk8 cells in presence of absence of Dox; Histone H3 (bottom) is used as loading control. **(C-G)** Combined PRC2 immunofluorescence with *Xist* RNA FISH (red) for **(C)** *Cdk8* WT transgene, **(D-E)** *Cdk8* D151A mutant transgene, and **(F-G)** *Cdk8* ΔATP mutant transgene complemented ΔCdk8 cells. DNA was stained with DAPI (blue). Scale bar: 5μm.

Development • Supplementary information

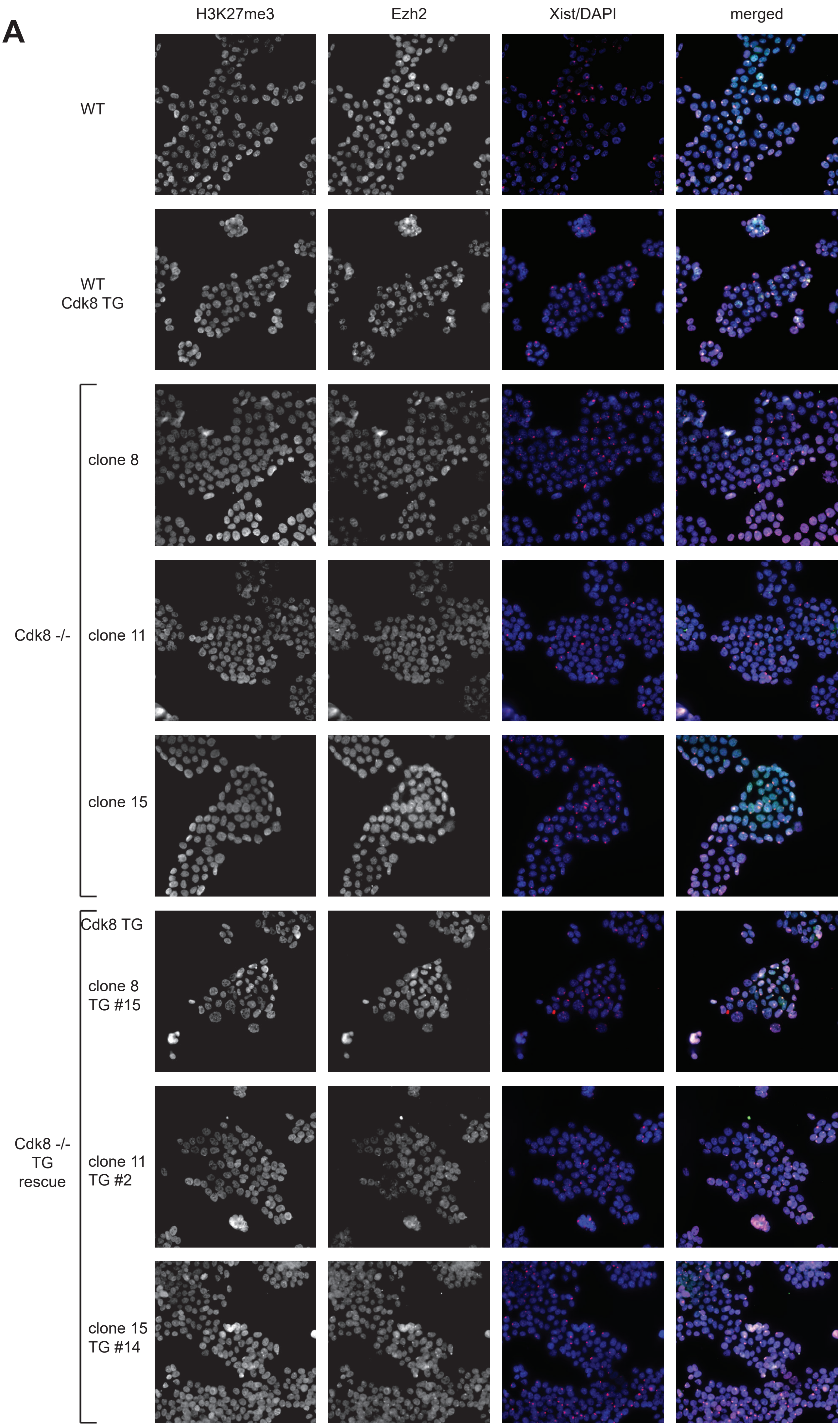
A
H3K27me3
Ezh2
Xist/DAPI
merged
WT
WT
Cdk8 TG
Cdk8 -/-
clone 8
clone 11
clone 15
Cdk8 TG
clone 8
TG #15
Cdk8 -/-
TG
rescue
clone 11
TG #2
clone 15
TG #14

Figure S4

B

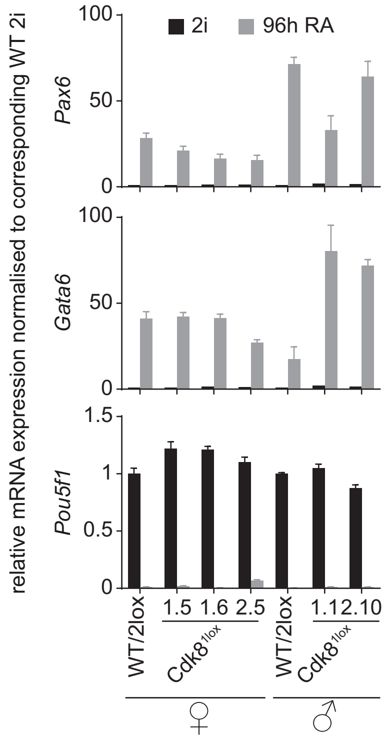

2i
96h RA
relative mRNA expression normalised to corresponding WT 2i
Pax6
Gata6
Pou5f1
100
50
0
1.5
1
0.5
WT/2lox
1.5 1.6 2.5
Cdk8 1lox
WT/2lox
1.1 2.10
Cdk8 1lox
♀
♂

C

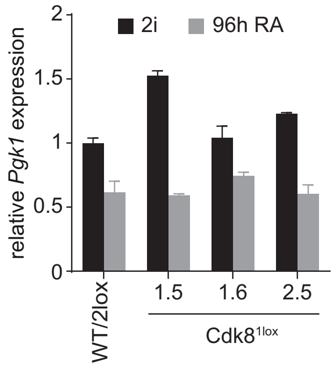

2i
96h RA
relative Pgk1 expression
2
1.5
1
0.5
0
WT/2lox
1.5
1.6
2.5
Cdk8 1lox

D

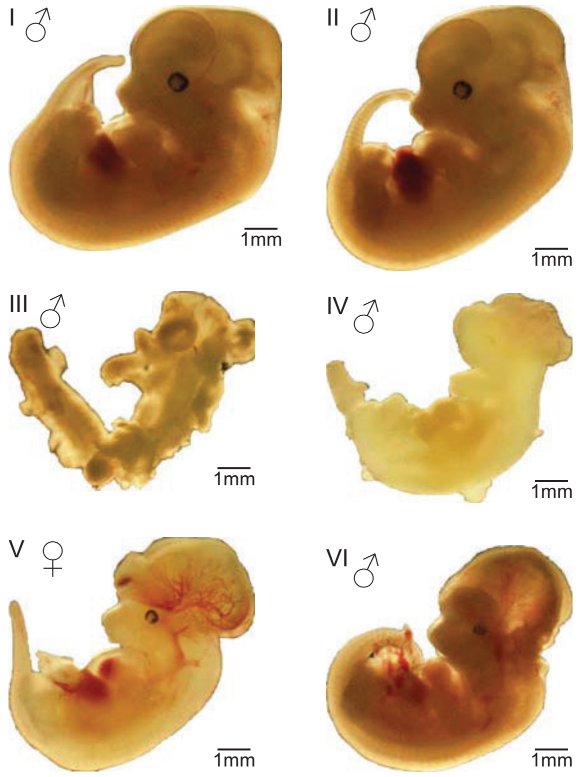

I ♂
II ♂
III ♂
IV ♂
V ♀
VI ♂
1mm

E

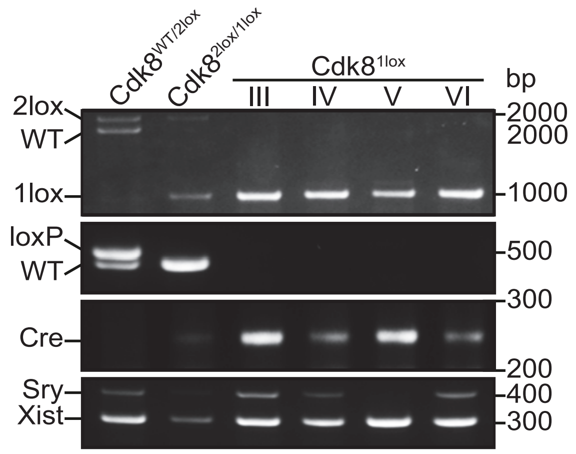

Cdk8 WT/2lox
Cdk8 2lox/1lox
Cdk8 1lox
III
IV
V
VI
bp
2lox
WT
1lox
loxP
WT
Cre
Sry
Xist
2000
2000
1000
500
300
200
400
300

Figure S4. *Cdk8* is required for efficient recruitment of PRC2. (A) Combined Ezh2 and H3K27me3 immunofluorescence (green), and *Xist* RNA FISH (red) images of wildtype, Cdk8 mutant, and rescued ESCs as indicated. A large field of view with several cells of individual channels and a merged image are shown for overview. DNA stained with DAPI (blue). **(B,C)** qRT-PCR expression analysis of RA induced differentiation in homozygous $Cdk8^{1lox}$ mutant and control wild type female ESCs. **(B)** Expression of *Pax6* (top), *Gata6* (middle), and *Pou5f1* (bottom) normalised to *Gapdh* and relative to WT levels is shown for undifferentiated ESCs in 2i medium (black bars) and after 96h RA differentiation (grey bars); experiment performed in triplicates; error bars represent SD. **(C)** *Pgk1* repression in 2i conditions (black bars), and after 96h RA differentiation (gray bars) normalised to Gapdh and relative to WT; experiment performed in triplicates; error bars represent SD. **(D)** Representative pictures of E12.5 embryos, scale bar: 1mm; I: $Cdk8^{WT/2lox}$, II: $Cdk8^{WT/2lox}$ Sox2-Cre embryos carry a heterozygous, and III-VI: $Cdk8^{1lox/1lox}$ Sox2-Cre embryos carry a homozygous *Cdk8* mutation. **(E)** PCR analysis of genotypes of embryos in panel D. Deletion PCR (top): 1lox, 2lox, and WT product sizes as indicated; (below) integration of the loxP site of $Cdk8^{2lox}$ and WT product sizes as indicated; (below) Cre recombinase transgene PCR; Sex specific PCR (bottom): Sry, and Xist product sizes as indicated.

Development • Supplementary information

Figure S5

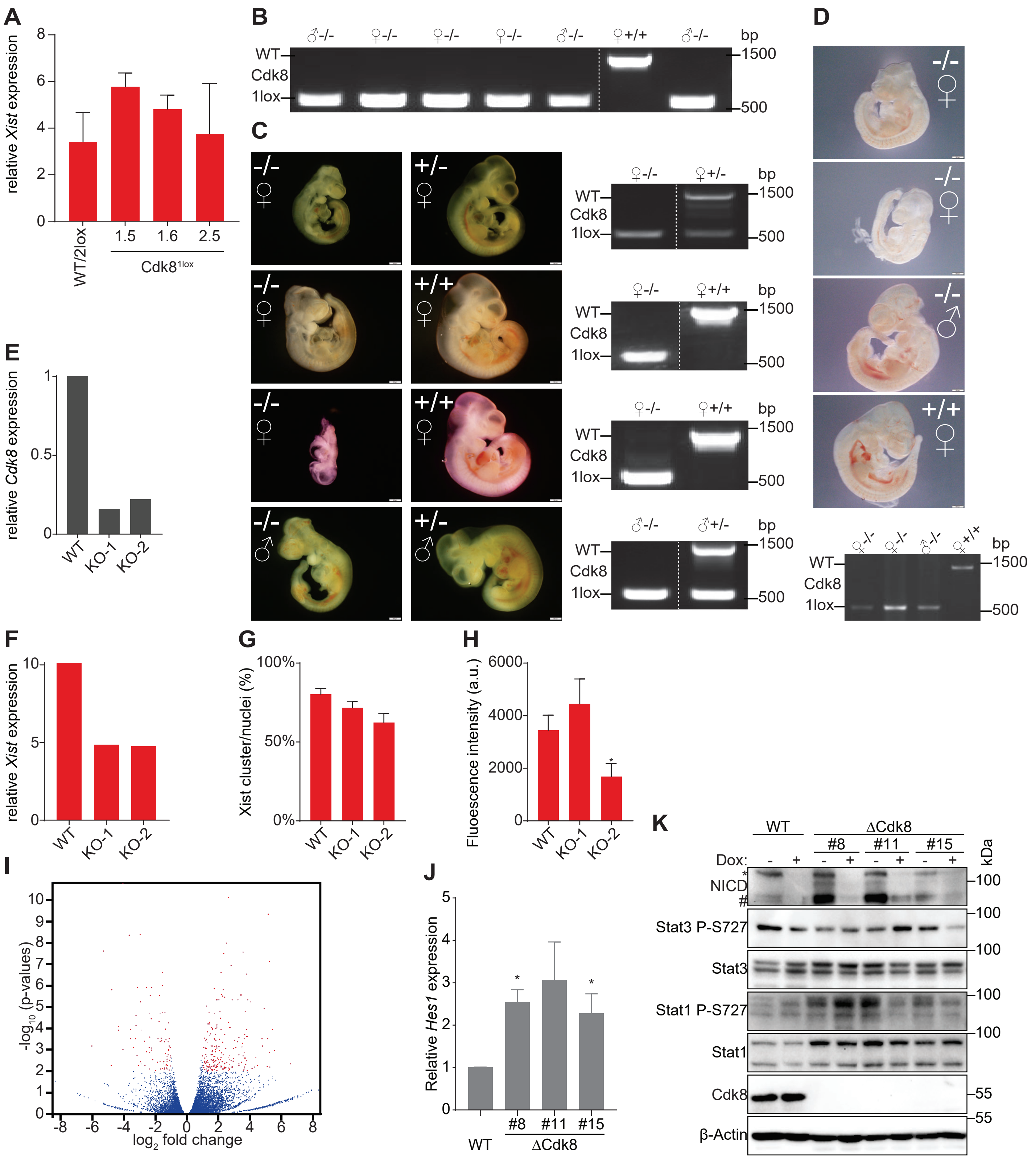

Development • Supplementary information

Figure S5. *Cdk8* contributes to the initiation of XCI. (A) *Xist* expression in $Cdk8^{1lox}$ ES cells; normalised to *Gapdh* and relative to 2i conditions; experiment performed in triplicates; error bars represent SD. **(B)** Gel images from *Cdk8* PCR genotyping of embryos shown in Fig. 4F with WT (upper) and 1lox fragments (lower); horizontal white dashed line indicates cropped image. **(C)** Images of homozygous $Cdk8^{1lox/1lox}$ mutant embryos (left) with corresponding wildtype $Cdk8^{+/+}$ or heterozygous $Cdk8^{+/1lox}$ control embryos (right) from the same litter; scale bar 500µm. PCR validation of $Cdk8^{1lox/1lox}$ genotype; upper band represents wild type DNA, lower band represents deletion band; horizontal white dashed line indicates cropped image. **(D)** Representative images of E10.5 embryos used for MEF derivation. Two $Cdk8^{1lox/1lox}$ female (top), one $Cdk8^{1lox/1lox}$ male (below), and one $Cdk8^{+/+}$ female (bottom) embryo are shown; scale bar represents 500µm. **(E-F)** qRT-PCR analysis of **(E)** *Cdk8* and **(F)** *Xist* expression in female primary mouse embryonic fibroblasts derived from E10.5 $Cdk8^{1lox/1lox}$ embryos; normalised to *Gapdh* and relative to female wildtype MEF; experiment performed once. **(G)** Quantification of *Xist* clusters from RNA FISH analysis. Percentages are relative to counted nuclei (>100 counted); experiment performed in triplicates; error bars represent SD. **(H)** *Xist* RNA FISH intensity quantification; >100 cluster measured; experiments performed in triplicates; error bars represent SD; asterisk indicates significant change relative to WT (*P*-value<0.05). **(I)** Volcano blot showing differentially expressed genes in ΔCdk8 relative to WT ESCs. Significantly deregulated genes (red) are defined by a two-fold change, and FDR *P*-value<0.01. **(J)** *Hes1* expression in WT and ΔCdk8 ESCs normalised to *Gapdh* and relative to WT expression levels; experiments performed in triplicates; error bars represent SD; asterisk indicates significant changes relative to WT (*P*-value<0.05). **(K)** Immunoblot for known *Cdk8* targets; from top: NICD: Notch intracellular domain (#: NICD, *: Notch transmembrane domain); Stat3 P-S727: phosphorylated Stat3 serine727; Stat3: total Stat3; Stat1 P-S727: phosphorylated Stat1 serine727; Stat1: total Stat1; Cdk8: confirmation of Cdk8 depletion, β-Actin: loading control.

Development • Supplementary information

Figure S6

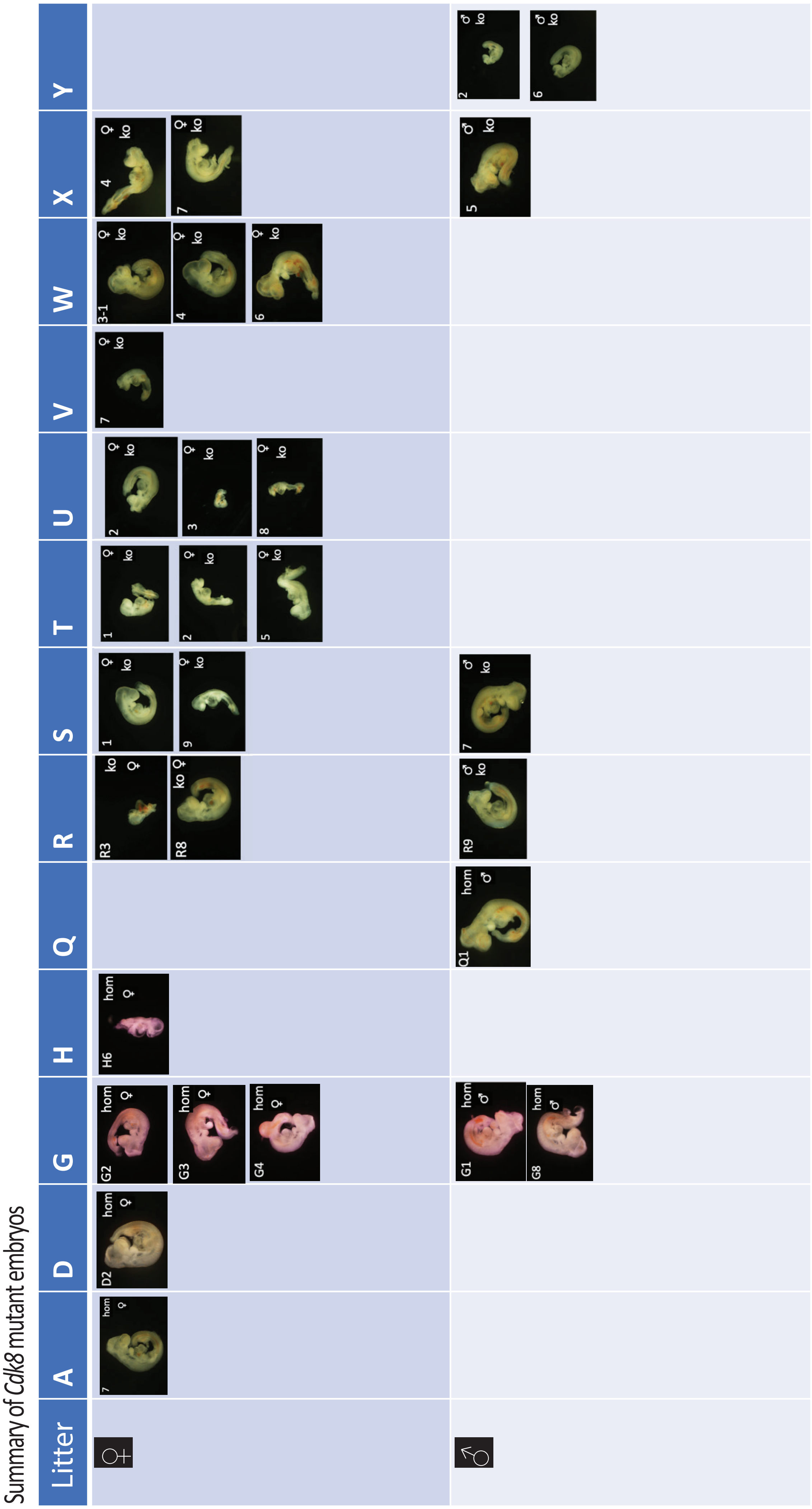
Summary of Cdk8 mutant embryos
Litter
A
D
G
H
Q
R
S
T
U
V
W
X
Y
hom
ko

Figure S6. Summary of *Cdk8* mutant embryos. Overview of microscopy images of homozygous *Cdk8* mutant embryos organized according to litters and separated according to sex. These images were used together with images of littermates for image segmentation and statistical analysis of embryo sizes in Fig. 4G.

Development • Supplementary information

Figure S7

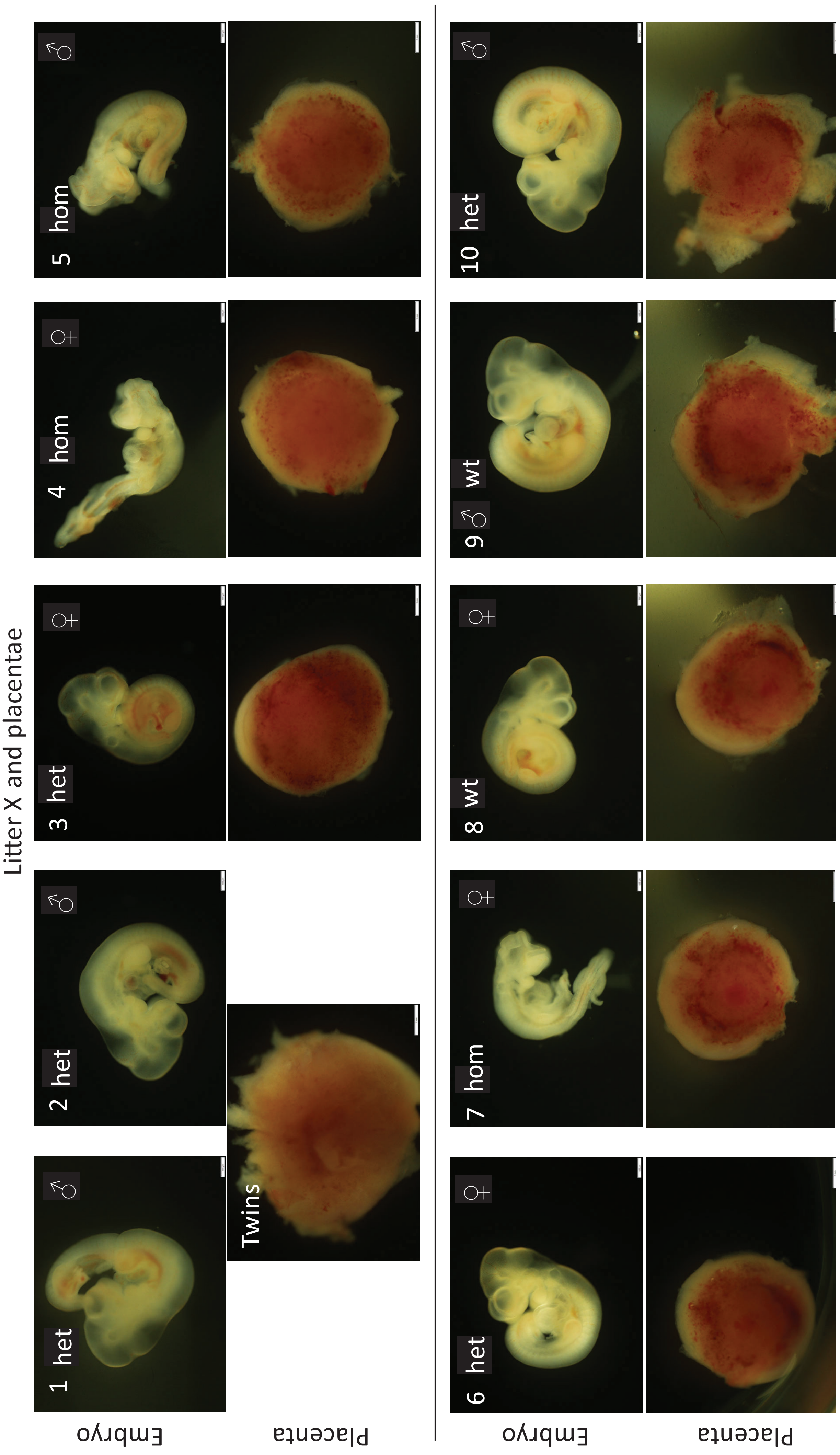
Litter X and placentae
Embryo
Placenta
1 het
2 het
3 het
4 hom
5 hom
Twins
6 het
7 hom
8 wt
9 wt
10 het

Figure S7. Microscopy images of *Cdk8* mutant embryos and placentae. Images of embryos (scale bar : 500 µm) and their placentae (scale bar : 1mm) are shown for litter X. Genotypes and sex of the embryos is indicated. Note that embryo 1 and 2 were twinned and shared one placenta.

Development • Supplementary information
